# Transient commensal clonal interactions can drive tumor metastasis

**DOI:** 10.1038/s41467-020-19584-1

**Published:** 2020-11-16

**Authors:** Suha Naffar-Abu Amara, Hendrik J. Kuiken, Laura M. Selfors, Timothy Butler, Marco L. Leung, Cheuk T. Leung, Elaine P. Kuhn, Teodora Kolarova, Carina Hage, Kripa Ganesh, Richard Panayiotou, Rosemary Foster, Bo R. Rueda, Athena Aktipis, Paul Spellman, Tan A. Ince, Joanne Xiu, Matthew Oberley, Zoran Gatalica, Nicholas Navin, Gordon B. Mills, Rodrick T. Bronson, Joan S. Brugge

**Affiliations:** 1 Department of Cell Biology, Harvard Medical School, Boston, MA 02115 USA; 2 Department of Molecular and Medical Genetics, Oregon Health & Science University Portland, Portland, OR 97239-3098 USA; 3 Department of Genetics, University of Texas MD Anderson Cancer Center, Houston, TX 77030 USA; 4 Vincent Center for Reproductive Biology and Division of Gynecologic Oncology, Department of Obstetrics and Gynecology, Massachusetts General Hospital, Boston, MA 02114 USA; 5 Harvard Medical School, Boston, MA 02115 USA; 6 Arizona Cancer Evolution Center and Department of Psychology, Arizona State University, Tempe, AZ 85281 USA; 7 Department of Pathology and Laboratory Medicine, Weill Cornell Medicine, New York, NY USA; 8 New York Presbyterian-Brooklyn Methodist Hospital, Brooklyn, NY 11215 USA; 9 Caris Life Sciences, Phoenix, AZ 85040 USA; 10 Department of Cell, Developmental and Cancer Biology, Oregon Health and Science University Knight Cancer Institute, Portland, OR 97239-3098 USA; 11 Rodent Histopathology Core, Harvard Medical School, Boston, MA 02115 USA; 12 Present Address: Cancer, Ageing and Somatic Mutation, Wellcome Trust Sanger Institute, Hinxton, UK; 13 Present Address: The Center for Applied Genomics, Children’s Hospital of Philadelphia, Pennsylvania, PA 19104 USA; 14 Present Address: Department of Pharmacology, Masonic Cancer Center, University of Minnesota Medical School, Minneapolis, MN 55455 USA; 15 Present Address: Department of Medicine, Dartmouth-Hitchcock Medical Center, Lebanon, NH 03766 USA; 16 Present Address: Department of Obstetrics & Gynecology, University of Washington, Seattle, WA 98195 USA; 17 Present Address: Roche Innovation Center Munich, Roche Pharmaceutical Research and Early Development, Nonnenwald 2, 82377 Penzberg, Germany; 18 Present Address: Meyer Cancer Center, Weill Cornell Medicine, New York, NY USA; 19 Present Address: The Biochemistry, Structural, Developmental, Cell and Molecular Biology Allied PhD Program, Weill Cornell Medicine, New York, NY 10065 USA; 20 Present Address: Department of Pathology, University of Oklahoma Health Sciences Center, Oklahoma City, OK 73104 USA

**Keywords:** Tumour heterogeneity, Mechanisms of disease, Phylogenetics, Ovarian cancer, Metastasis

## Abstract

The extent and importance of functional heterogeneity and crosstalk between tumor cells is poorly understood. Here, we describe the generation of clonal populations from a patient-derived ovarian clear cell carcinoma model which forms malignant ascites and solid peritoneal tumors upon intraperitoneal transplantation in mice. The clonal populations are engineered with secreted *Gaussia* luciferase to monitor tumor growth dynamics and tagged with a unique DNA barcode to track their fate in multiclonal mixtures during tumor progression. Only one clone, CL31, grows robustly, generating exclusively malignant ascites. However, multiclonal mixtures form large solid peritoneal metastases, populated almost entirely by CL31, suggesting that transient cooperative interclonal interactions are sufficient to promote metastasis of CL31. CL31 uniquely harbors *ERBB2* amplification, and its acquired metastatic activity in clonal mixtures is dependent on transient exposure to amphiregulin, which is exclusively secreted by non-tumorigenic clones. Amphiregulin enhances CL31 mesothelial clearance, a prerequisite for metastasis. These findings demonstrate that transient, ostensibly innocuous tumor subpopulations can promote metastases via “hit-and-run” commensal interactions.

## Abstract (web summary)

Cooperative interactions among tumor cells may have important implications for metastasis. Here, the authors examined the spatio-temporal nature of interactions among clonal populations of ovarian carcinoma cells and found that transient interactions cells can promote metastases via commensal interactions.

## Introduction

Tumors consist of diverse subpopulations of neoplastic cells which contribute to intratumoral heterogeneity^1^. Studies have suggested that genetically and epigenetically heterogeneous subpopulations possess diverse functional abilities^2,3^, however, the consequences of this intratumoral functional diversity on behavior of the tumor as a whole is poorly understood. The classical Darwinian model of tumor evolution posits that genetic variants generated during tumor progression compete and, in time, progressively displace one another^4–6^. However, the long-term maintenance of coexisting genetically distinct subpopulations within the same tumor suggests that cooperation between distinct subpopulations likely influences the tumor phenotype—that is, where one subpopulation provides an essential biological function that is required by others within the tumor, and thus influences the overall composition and behavior of the tumor.

Several studies in mouse models and in *Drosophila* have demonstrated that subpopulations of cells can cooperate to induce tumor growth^7–9^ and metastasis^10–15^. In diffuse intrinsic pontine glioma, Vinci et al.^16^ identified a cooperative mechanism between H4K20 methyltransferase-wild-type and -mutant subpopulations that promotes invasion. In all of the aforementioned studies, either specific tumor subpopulations with pre-defined markers or genetically engineered subclonal populations were examined. Functional studies of intratumoral cooperation during tumor progression using a collection of patient-derived clonal populations without bias toward a specific marker has not been reported.

Multiple studies have tracked clonal populations in the context of tumor progression. Kerso et al.^17,18^ examined the fate of lentiviral-tagged populations of colon tumor cells during tumorigenesis and demonstrated that the representation of clonal populations changes over time. Using genetic lineage tracing, Driessens et al.^19^ identified two distinct groups of clones with different proliferation and renewal potential, providing experimental evidence for the existence of cancer stem cells in unperturbed solid tumor growth. However, it was not feasible to address the mechanisms underlying the observed clonal dynamics described in these reports since the clones could not be isolated for mechanistic studies.

Research suggests that cooperative interactions among tumor cells may have important implications for metastasis. For example, Aceto et al.^20^ discovered that circulating clusters of multiclonal tumor cells were more effective at metastasizing than single circulating tumor cells in a mouse model, and that these clusters were more resistant to apoptosis than single cells. They also demonstrated that, in patients, higher levels of cell adhesion molecules (plakoglobins) were associated with poorer outcomes. Chapman et al.^21^ similarly discovered that multiclonal tumor cell groups produce extracellular matrix components and proteases that are associated with greater invasiveness. These results suggest that cooperation among cancer cells is likely important during invasion and metastasis, but leaves many open questions about the potential mechanisms of molecular crosstalk that underlie this cooperation and how they change over time.

Here, we describe the generation of a collection of single-cell clonal populations from a patient-derived clear cell carcinoma (CCC) cell line, OCI-C5x^22^. We then selected a panel of 11 clones based on their heterogenous morphology and rates to confluence in culture, and tracked their growth dynamics in vivo by assessing Gaussia luciferase activity in blood and characterized the tumorigenicity of each individual clonal population alone or in multiclonal mixtures. By “tagging” each clonal population with a unique DNA barcode, we monitored the clonal dynamics within tumors derived from multiclonal mixtures^18^. Our findings identify a commensal mechanism of clonal cooperation involving a transient interclonal interaction that promotes metastasis of one clonal population without benefiting the other.

## Results

### Isolation of single-cell clonal populations from a patient-derived CCC model

To investigate whether subpopulations within an individual tumor cooperate to affect tumor behavior as a whole we sought to systematically characterize the tumorigenicity and clonal growth dynamics of individual as well as mixtures of tumor subpopulations in vivo. To that end, we isolated single-cell clonal populations from OCI-C5x, an ovarian CCC cell line generated from a patient-derived xenograft (PDX) of a treatment-naïve patient primary tumor. OCI-C5x line maintains the spectrum of genetic alterations of the original patient tumor and, when injected into the peritoneum of immunocompromised mice, generates tumors that recapitulate the histopathological features and molecular markers of CCC and of the original tumor^22^. To facilitate functional studies and monitoring of tumor burden in mice over time, OCI-C5x cells were engineered to co-express the fluorescent marker tdTomato and Gaussia luciferase (Gluc), a secreted form of luciferase that makes it feasible to track tumor burden by assaying whole blood luciferase activity^23^. Single OCI-C5x cells were sorted into wells of a 384-well plate and cultured with parental OCI-C5x-condition media to support their initial expansion (Supplementary Fig. 1A). The plates were inspected under a fluorescence microscope four hours after sorting and wells containing more than one cell were flagged and excluded from further analysis. Out of 90, 43 single cells expanded to generate clonal populations. From these, we chose 11 clones (CL09, CL11, CL12, CL16, CL17, CL28, CL31, CL41, CL44, CL46, and CL49) based on varied morphology in culture (Supplementary Fig. 1B) and time to confluence (range: 7–9 weeks) for further characterization.

### Clonal populations exhibit variable growth dynamics in vivo

**Fig. 1:**
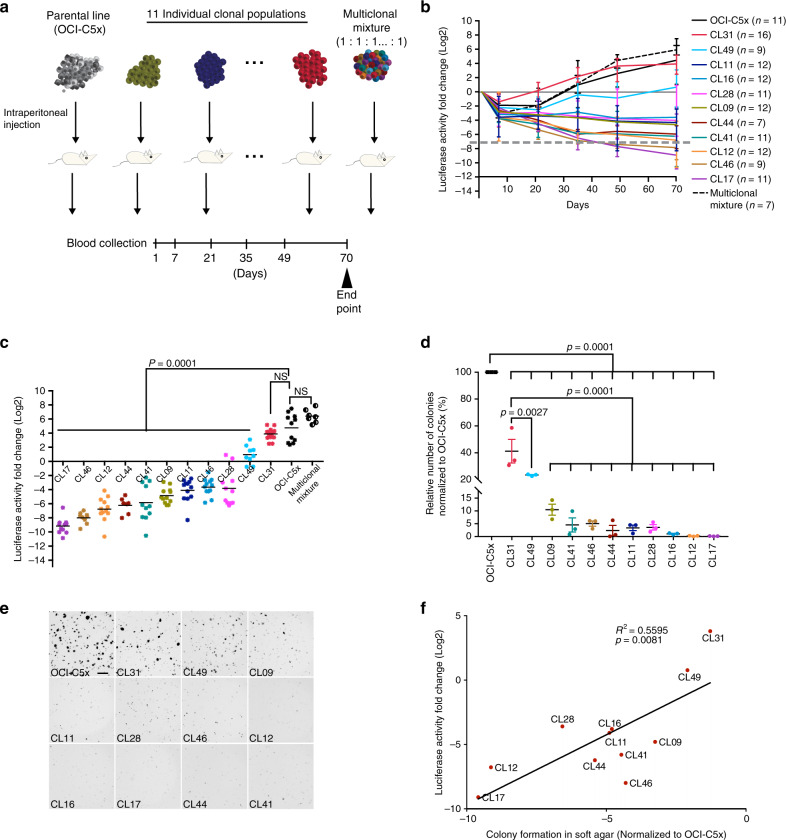
Clonal populations display variable growth dynamics in vivo. **a** Schematic depiction of tumor cell populations inoculated into immunocompromised (NSG) female mice. **b** Tumor growth dynamics in vivo assessed by measurement of luciferase activity in whole blood samples collected at the indicated time points. Dashed line represents Gluc values at background levels. Data presented as fold change in luciferase activity compared to 24 h post injection. Data represent mean ± SD of each group (*n* = as indicated) from three independent experiments. **c** Fold change in luciferase activity per mouse determined as in (**b**) at the 10-week end point. *p* Values were computed using the one-way ANOVA test followed by Dunnett’s multiple comparison test, comparing parental line to each of the clones and the multiclonal mixture. NS not significant. **d** Colony formation in soft agar measured after 21 days and normalized to the parental line. Data represent mean ± SEM from three independent experiments, biological replicates were performed on cells that had been passaged anywhere between 8 and 23 times. *p* Values were computed using the one-way ANOVA test followed by Dunnett’s multiple comparison test, comparing either parental or CL31 against each of the clones. **e** Images of a representative soft agar colony formation assay from one of three independent experiments. Scale bar represents 1 mm. **f** Linear regression analysis of colony formation in soft agar and tumor burden (measured by luciferase activity in blood samples collected at 10-week end point) for all 11 clonal populations. *p* = 0.0081, two-tailed. See also Supplementary Figs. 1–3.

To assess the ability of each individual clonal population to grow after intraperitoneal implantation into immunocompromised mice (referred to as tumorigenicity in this system) and compare it to that of the OCI-C5x parental line or of a heterogeneous mixture of the clonal populations, we injected equal cell numbers (3 × 10^6^) of each individual clonal population, the OCI-C5x line, or a defined heterogeneous mixture consisting of all 11 clones in equal proportion (referred to as multiclonal mixture) into the peritoneum of immunocompromised female mice (NOD *scid* gamma-NSG) (Fig. 1a). Total tumor burden was quantified over time via assessment of Gluc activity in the blood^23^ (Fig. 1b). We confirmed that the Gluc levels correlate with cell number in vivo in this model (*R*^2^ = 0.9949, *P* = 0.0015) (Supplementary Fig. 2). We found that although all the populations exhibited a dramatic cell loss in the first week following injection, their recovery and growth rate varied (Fig. 1b, c). Both CL31 and the multiclonal mixture displayed recovery and growth rates that were comparable to the OCI-C5x parental line. CL49 persisted with a lower tumor burden than the parental line and only moderately grew during the duration of the experiment. CL09, CL11, CL12, CL16, CL28, CL41, and CL44 persisted with minimal viability following the initial period of cell loss, but failed to increase in cell number within the experimental time frame. The two remaining clonal populations (CL17 and CL46) exhibited continuous cell loss over time and the Gluc levels in blood were at background levels around the 5-week time point on average (Fig. 1b). These results indicate that, with the exception of CL31, and to a lesser extent CL49, the majority of the clonal populations had minimal to no tumor-forming activity. These results were reproducible across multiple independent experiments performed in a span of over 2 years, suggesting that this phenotypic diversity is driven by stable cell autonomous properties.

To investigate whether the tumorigenicity of the clonal populations could be attributed to enhanced proliferative capacity, we assessed the correlation between in vitro doubling time in monolayer culture and tumor growth (Gluc levels) (Supplementary Fig. 3A, B). We found that tumorigenicity did not correlate with in vitro proliferation rates (*R*^2^ = 0.1248, *P* = 0.3763). Next, we examined the ability of the clonal populations to form colonies in soft agar, a commonly used surrogate for tumor formation. Among the clonal populations, CL31 formed the most colonies followed by CL49, and the remaining clones formed few, if any, colonies. This in vitro phenotypic diversity was stable across three independent experiments, performed with cells passaged between 8 and 23 times. Tumorigenicity correlated with colony formation in soft agar (*R*^2^ = 0.5595, *P* = 0.0081) (Fig. 1d–f), suggesting that anchorage independence reflects tumorigenicity in our model system.

### Individual clonal populations fail to form solid peritoneal metastases

**Fig. 2:**
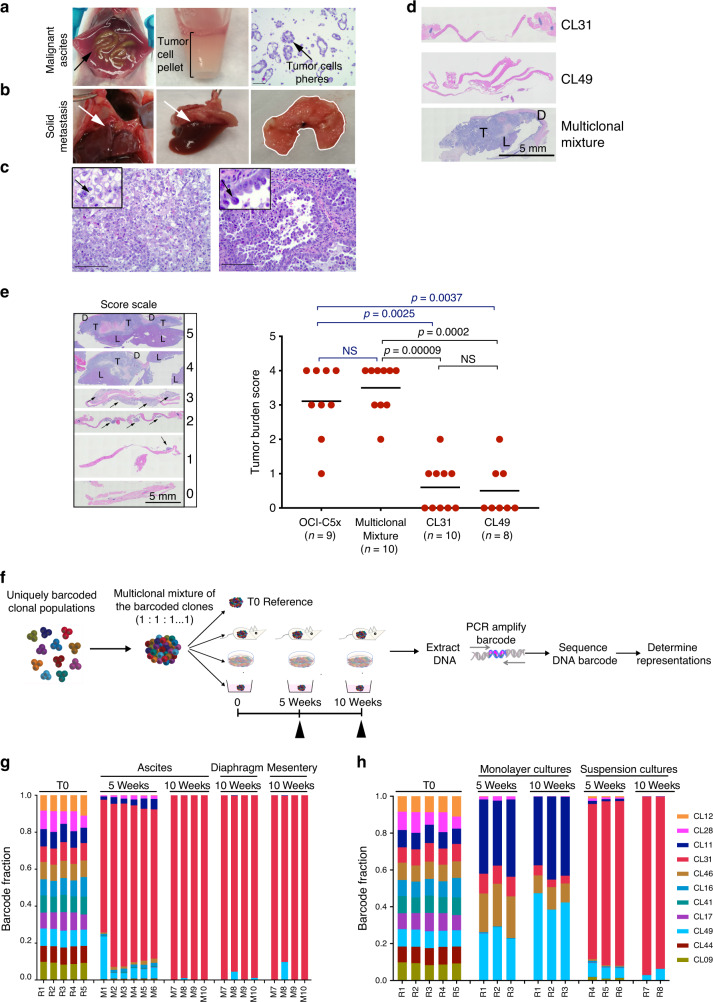
Individual clonal populations fail to form solid peritoneal metastases. **a** Representative images of OCI-C5x generated malignant ascites, and **b** solid tumors on the diaphragm, which connect to the liver (white arrow). **c** H&E-stained sections from the solid tumor masses, representative images from one of three independent experiments. Note that the tumor cells maintain histopathological characteristics of CCC in patients with “clear” cells (C-left image, arrow) and hobnail features (C-right image, arrow). Scale bar, 100 μm. **d** Representative H&E images of tumor sections of solid metastases on diaphragm generated by CL31, CL49, and the multiclonal mixture, representative images from one of two independent experiments. T tumor cells, D diaphragm, L liver. Scale bar, 5 mm. **e** Tumor burden score of solid metastases on the diaphragm (data from two independent experiments, *n* = 8–10 mice per group). Scored blindly. *p* Values were computed using the chi-square test with Monte Carlo simulation. **f** Schematic depiction of experimental design and workflow for barcode experiments. **g** Barcode representation in the indicated samples collected from mice injected with the multiclonal mixture composed of barcoded clones, and **h** in samples collected from monolayer and suspension cultures in vitro. A representative of two independent experiments.

Ovarian carcinoma metastatic dissemination is distinct from that of other solid cancers in that it is primarily non-hematogenous and limited to the peritoneal cavity, where tumor cells, once exfoliated from the primary site, are spread throughout the abdominal cavity and grow in the peritoneal fluid as small clusters (malignant ascites) and form solid peritoneal masses on the mesothelial surfaces, with clear preferences for the omentum and the diaphragm^24^. The pattern of growth of the OCI-C5x cell line in immunocompromised mice recapitulates these disease features, consistently generating the two forms of tumor cell growth: malignant ascites, with 400–1000 μl of cell pellet volume (Fig. 2a), as well as solid peritoneal metastases on the mesentery, ovary, and large tumors on the diaphragm that often extend to the liver (Fig. 2b). Moreover, OCI-C5x tumors exhibit “clear” and “hobnail” cells, typical CCC histopathological features (Fig. 2c). Consistent with the blood Gluc assay results, only CL31, CL49, and the multiclonal mixture produced any detectable tumor cell growth in immunocompromised mice. CL49 displayed weak in vivo cell growth, generating only 10–50 μl cell pellet volume of malignant ascites, whereas CL31, like the OCI-C5x parental line, consistently generated more than 400 μl (ranges between 400 and 1000 μl) cell pellet volume of malignant ascites. However, unlike OCI-C5x, neither CL31 nor any of the other clones generated macroscopically detectable solid peritoneal metastases. In contrast, the multiclonal mixture fully recapitulated the phenotype of the parental line, generating robust malignant ascites (>400 μl cell pellet volume) as well as solid peritoneal metastases that were most prominent on the diaphragm and often extended to the liver (Fig. 2d). Blind scoring of tumor burden in the diaphragm histology sections from mice injected with CL31, CL49, OCI-C5x, or the multiclonal mixture confirmed that CL31 and CL49 failed to recapitulate the solid peritoneal metastasis phenotype of the OCI-C5x parental line or the multiclonal mixture (Fig. 2e). These data strongly suggest that the OCI-C5x-derived clonal populations cooperate to generate solid peritoneal metastases, a phenotype that the individual populations are incapable of producing on their own.

### Malignant ascites and solid peritoneal metastases derived from the multiclonal mixture are dominated by CL31

To identify the clonal representation within the malignant ascites and solid peritoneal metastasis generated by the multiclonal mixture, we tagged each clonal population with a unique semi-random 30 base-pair DNA barcode with balanced GC content (50%) to ensure uniform polymerase chain reaction (PCR)-amplification efficiency across the barcodes^18^. To spatially and temporally detect the representation of each clonal population during tumor progression in vivo, malignant ascites were collected at 5 and 10 weeks after IP injection of the multiclonal mixture and solid peritoneal metastasis at 10 weeks. In parallel, we included two in vitro arms: (1) standard monolayer culture of the multiclonal mixture passaged every four days, and (2) suspension culture of the multiclonal mixture in which media was refreshed every 4 days. The genomic DNA of all tumor samples and cell cultures collected at 5 and 10 weeks was extracted, and the barcode sequences were PCR-amplified and subjected to next generation sequencing (NGS). The barcode representations were compared to that of the initial (T0) multiclonal mixture (Fig. 2f). The barcode representation analyses indicated that, at the 5-week time point, the malignant ascites consisted of multiple clonal populations, with a clear dominance of CL31 (82.1% on average); other clones were present at lower frequencies (predominantly CL49, with lesser representation by CL11, CL28, CL16, and CL46 and trace amounts of CL09, CL12) (<30% combined). At the 10-week time point, the tumor samples consisted almost entirely of CL31 (>97% at all sites) (Fig. 2g) and the relative abundance of the other clones dropped below 0.0001% at all sites, with exception of CL49 (0.25% in ascites, 1.25% diaphragm, and 2.25% mesentery on average). The presence of the minor clones at the five-week time point was consistent with the tumor-forming ability of these specific clones as they maintained minimal viability when injected individually (Fig. 1b). Moreover, the dominant representation of CL31 in the malignant ascites at five and ten weeks was in agreement with this clone’s individual, robust tumor-forming capacity (Fig. 1b). However, the dominance of CL31 in the solid peritoneal tumors on the mesentery and diaphragm was unexpected since no solid peritoneal metastases were detectable when CL31 alone was injected IP (Fig. 2e). CL49, the second most tumorigenic clone, dominated both ascites and solid metastasis on diaphragm and mesentery when CL31 was removed from multiclonal mixtures (Supplementary Fig. 4).

Analysis of the clonal representation in the multiclonal mixture of all clones in vitro indicated that the dominance of CL31 was not recapitulated in monolayer cultures where CL31 was outcompeted at early time points by CL11, CL28, CL46, and CL49 (Fig. 2h), suggesting that the dominance of CL31 in vivo is not due to superior proliferative fitness in the clonal mixture. In contrast to the monolayer culture clonal representation, the pattern of clonal dynamics observed under suspension conditions in vitro largely recapitulated that observed in vivo, with CL31 outcompeting the rest of the clones as early as the 5-week time point. These results are in line with the anchorage independence of this clone and are consistent with anchorage-independent survival being associated with the competence to grow in vivo in ascites fluid. These results, together with the finding that CL31 is limited to generation of malignant ascites and is unable to produce solid peritoneal metastases on its own, provide strong evidence that interclonal cooperation is required to promote the acquisition of solid peritoneal metastasis activity by CL31.

### Genetic analyses identify differential *ERBB2* amplification in CL31

**Fig. 3:**
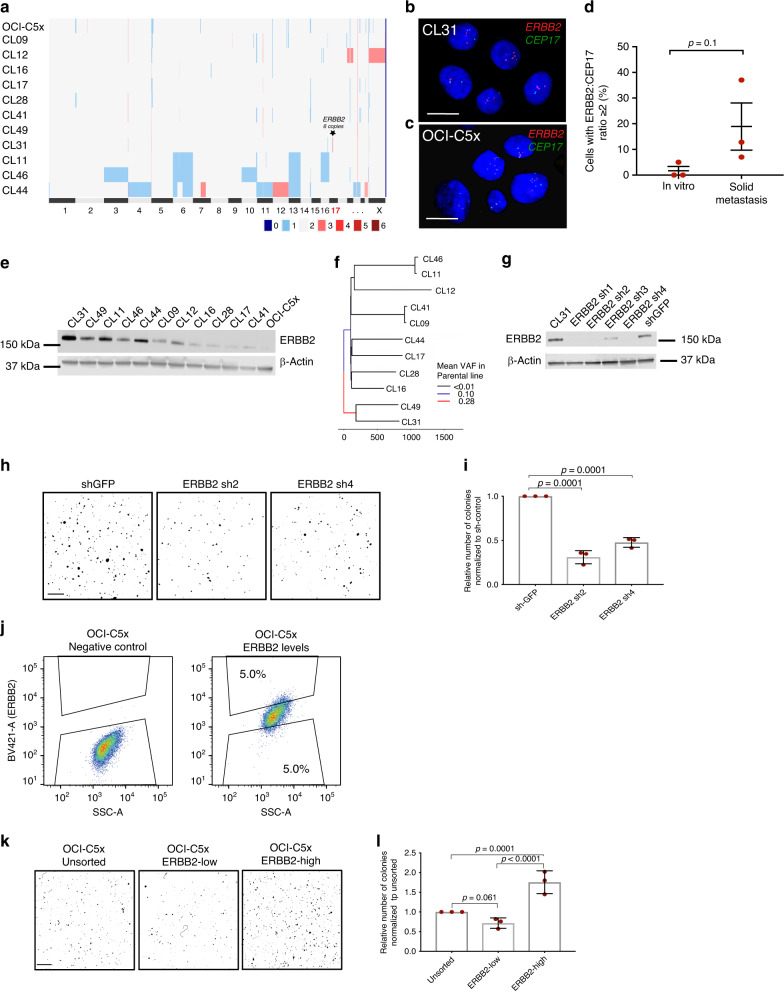
Molecular analyses of clonal populations reveal CL31-specific genetic alterations. **a** Heatmap representation of copy number alterations in the parental OCI-C5x cell line and clonal populations. **b**, **c** Representative images from one of three independent experiments of FISH staining for *ERBB2* (red) and CEP17 (green) of **b** CL31 and **c** parental OCI-C5x cells in vitro. Scale bar 20 μm. **d** Quantification of cells with *ERBB2:CEP17* copy number ratio equal or greater than two (FISH data) in the OCI-C5x cell line in vitro from experiment in (**b**) and in solid metastases derived from the OCI-C5x cell line. Data shown as mean ± SEM from three independent experiments, *p* Value was computed using the Mann–Whitney test, two-tailed. **e** Representative Western blot of ERBB2 protein in the OCI-C5x cell line and each of the clonal populations. β-actin was used as a loading control, representative blots from one of two independent experiments. **f** Maximum parsimony tree generated from all filter-passing substitutions detected by whole-exome sequencing of all single-cell derived clonal populations, and an autologous DNA sample from the patient’s blood. Branches colored by mean VAF of mutations in parental clone. **g** Western blot showing *ERBB2* knockdown in CL31 using four distinct shRNAs, representative blots from one of three independent experiments. **h** Representative images and **i** quantification of colony formation in soft agar by CL31 expressing a control shRNA (GFP) or one of two distinct *ERBB2* shRNAs (Sh2 and Sh4). Scale bar, 500 μm. The number of colonies were normalized to that of shGFP control. Three independent experiments were summarized by mean ± SEM. *p* Values were computed using the one-way ANOVA test and corrected for multiple comparisons using the Dunnett’s method. **j** OCI-C5x parental cells were sorted into ERBB2-low and ERBB2-high expressing populations by FACS. **k** Representative images of colony formation in soft agar by the unsorted OCI-C5x parental line and the ERBB2-low and ERBB2-high populations. Scale bar, 250 μm. **l** Quantification of colony formation in soft agar in (**k**) by the indicated OCI-C5x populations. Data were normalized to that of the unsorted parental line. Three independent experiments were shown as mean ± SEM. *p* Values were computed using the one-way ANOVA test and corrected for multiple comparisons using the Dunnett’s method.

To investigate the molecular mechanism(s) underlying this clonal cooperation, we performed multiple genomic analyses. Copy number aberration (CNA) profiling of the OCI-C5x parental line revealed that OCI-C5x is mainly diploid with no dominant regions of chromosomal deletions or amplification detected in bulk analysis (Fig. 3a). However, the genetic heterogeneity of this cell line became apparent when the clonal subpopulations were analyzed. For example, while most of the clones are diploid, CL11, CL44, and CL46 harbor whole chromosome loss (e.g., chr 3, 4, 6, 10, 11, 13, and 16) or gains (7, 12, and X), a phenomenon commonly observed in CCC^25,26^. Notably, CNA analysis also revealed a focal amplification of *ERBB2* exclusively in CL31 (the amplified region of chromosome 17 which contains six copies of *ERBB2* is marked with a star) (Fig. 3a). Fluorescence in situ hybridization (FISH) confirmed the *ERBB2* amplification in CL31. The majority of the cells (99%) had an *ERBB2*:chromosome 17 (centromere CEP17) ratio of two or more with an overall average ratio of 2.54 (Fig. 3b).

To test whether *ERBB2* amplification pre-existed in the original patient sample from which OCI-C5x was derived, we performed FISH analysis on histology section of the original primary tumor (ovary primary site). The sample was scored twice by two independent technicians at the Cytogenomics Core Laboratory (Brigham and Women’s Hospital, Boston, MA) for *ERBB2*:CEP17 ratio in ten different regions identified visually by a pathologist as tumor cell-enriched areas and confirmed by staining with S100A1, a marker that differentially identifies ovarian carcinoma cells from normal ovarian tissue^27^ (Supplementary Fig. 5A). In three regions (regions #3, #4, and #6), more than 20% (26%, 23.3%, and 25%, respectively) of cells displayed *ERBB2*:CEP17 signal ratio equal or greater than two (cells with only one CEP17 signal were excluded), with an average ratio of *ERBB2*:CEP17 signal of 2.96, 2.42, and 2.7, respectively. Immunohistochemistry confirmed that a subset of tumor cells within region #3 express elevated levels of ERBB2 protein (Supplementary Fig. 5B–E). These results demonstrate that *ERBB2* amplification was present in the original patient tumor and was not acquired during derivation of the OCI-5Cx cell line.

We also examined the abundance of ERBB2-amplified cells in the OCI-C5x parental line by FISH analysis. In one out of three experiments, 5% of 100 scored cells displayed an *ERBB2*:CEP17 signal ratio equal to two; in two additional experiments in which 100 cells were scored in each, none displayed an *ERBB2*:CEP17 signal equal or greater than two. These results indicate that cells with amplification of *ERBB2* are rare in the parental cell line. Conversely, solid peritoneal metastases generated by the parental line contained a higher percentage of cells with an *ERBB2*:CEP17 ratio equal or greater than two (12%, 37%, and 7% in three independent xenografts), with average *ERBB2*:CEP17 ratio of 2.52 ± 0.15 (Fig. 3c, d). While not statistically significant (*p* = 0.1), this shows a trend towards enrichment of cells with extra copies of *ERBB2* in the metastatic tumors, suggesting selection in vivo. Moreover, analysis of ERBB2 protein levels by Western blotting confirmed that, among all the clones and the OCI-C5x parental line, CL31 has the highest levels of this protein while the OCI-C5x parental line has the lowest levels (Fig. 3e). Taken together, these analyses revealed alterations that would have otherwise been masked in bulk analysis of the OCI-C5x population and indicate that the *ERBB2* amplification is present at a low frequency in the original tumor and the OCI-C5x parental line derived from it in vitro.

We also performed whole-exome sequencing of all clones, the OCI-C5x parental line, and an autologous DNA sample from the patient’s blood, which revealed an extremely high mutation burden (106 mutations per megabase) that is likely driven by mutations in the mismatch DNA repair gene *MSH2* and the DNA damage response gene *ATM*, shared across all samples. This is also supported by the finding that over half of mutations can be attributed to mutational signatures 6 and 20 which have been previously associated with mismatch repair gene defects^28^ (Supplementary Fig. 6A–C). In addition, we found no significant variation in the proportion of signatures present in any of the clones. Maximum parsimony-based phylogenetic analysis of whole-exome mutations indicated that CL31 and CL49, the only tumor-forming clones, were located on one branch of the maximum parsimony tree, suggesting that they are more closely related genetically to each other than to other clones (Fig. 3f). Indeed, although all of the clones are related, CL31 and CL49 are quite distant from the other clones on the parsimony tree. We also analyzed single nucleotide variation allele frequencies of mutations belonging to each branch of the tree in the OCI-C5x parental population. This revealed that the mutations shared by CL31 and CL49 are at a higher abundance in the parent than any other clones (Wilcoxon, *p* < 0.0001). This finding suggests that a large proportion of the OCI-C5x parental line is comprised of a common ancestor of these two clones. Moreover, these data imply that *ERBB2* amplification, which was present only in CL31, may be a later event acquired in a small subset of the original tumor population.

### Elevated ERBB2 levels contribute to anchorage-independent growth of CL31

*ERBB2* amplification in CL31, the only robust tumor-forming clone, was of particular interest because *ERBB2* amplification and overexpression are more common in CCC than in other ovarian cancer subtypes^29–31^. Moreover, reverse phase protein assay (RPPA) analysis comparing CL31 to the rest of the clones identified ERBB2 levels as significantly higher in CL31 (*p* = 1.14 × 10^−3^, Student’s *t* test false-discovery rate (FDR) corrected) (Supplementary Fig. 7A). To examine whether the elevated ERBB2 levels in CL31 contribute to its robust phenotype, we evaluated the effects of shRNA-mediated *ERBB2* downregulation on soft agar colony formation, which correlated with tumorigenic ability in vivo in our model (Fig. 1f). Two distinct hairpins that induced dramatic reduction in ERBB2 protein levels impaired CL31 colony formation by approximately 50% compared to the sh-GFP control (Fig. 3g–i), indicating that ERBB2 contributes to the colony forming activity of CL31.

Given that the OCI-C5x parental line has the lowest levels of ERBB2 (Fig. 3e) but displays robust anchorage independence, we also assessed the colony forming activity of OCI-C5x cells with different levels of expression of ERBB2. Using FACS, we sorted OCI-C5x for subpopulations with high ERBB2 (highest 5%) and low ERBB2 (lowest 5%) expression (Fig. 3j). Colony formation in soft agar of these two subpopulations was compared to that of the original unsorted OCI-C5x line. ERBB2^hi^ cells displayed twofold greater colony forming activity than the unsorted population, and threefold higher than the ERBB2^lo^ subpopulation, respectively (Fig. 3k, l); the ERBB2^lo^ subpopulation activity was only slightly lower than the unsorted parental (*p* = 0.061 N.S.). This finding provides additional evidence that ERBB2 promotes anchorage-independent growth and also suggests that other proteins, can contribute to this phenotypic activity in our model.

To identify other proteins that might contribute to anchorage independence, we downregulated the antiapoptotic protein BCL-X_L_ which is highly expressed in both CL31 and CL49, the only two tumor-forming clones (Supplementary Fig. 7A). Knockdown of BCL-X_L_ in CL31 (>80% protein KD) inhibited colony forming efficiency by approximately 75% (Supplementary Fig. 7B, C). Attempts to generate stable cell lines expressing shRNAs targeting both BCL-X_L_ and ERBB2 using multiple distinct shRNA vectors were unsuccessful, suggesting that loss of both proteins affects cell viability/protliferation. To address whether overexpression of BCL-X_L_ is sufficient to promote anchorage-independent growth in our model, we overexpressed BCL-X_L_ in CL11 and CL17, high and low ERBB2 expressing clones, respectively (Supplementary Fig. 7D, E). While overexpression of BCL-X_L_ enhanced the ability of these clones to grow in soft agar by twofold (*p* = 0.038 and *p* = 0.016, respectively), the colony forming efficiency of both was very low compared to CL31 since these two non-engineered clones have very weak basal colony forming activity. These findings suggest that while BCL-X_L_ and ERBB2 significantly contribute to the anchorage independence activity of CL31, their expression alone is not sufficient to confer the high level of anchorage independence associated with CL31 or other clones (e.g., CL11 and CL17).

### Overexpression of amphiregulin induces peritoneal metastasis of CL31

**Fig. 4:**
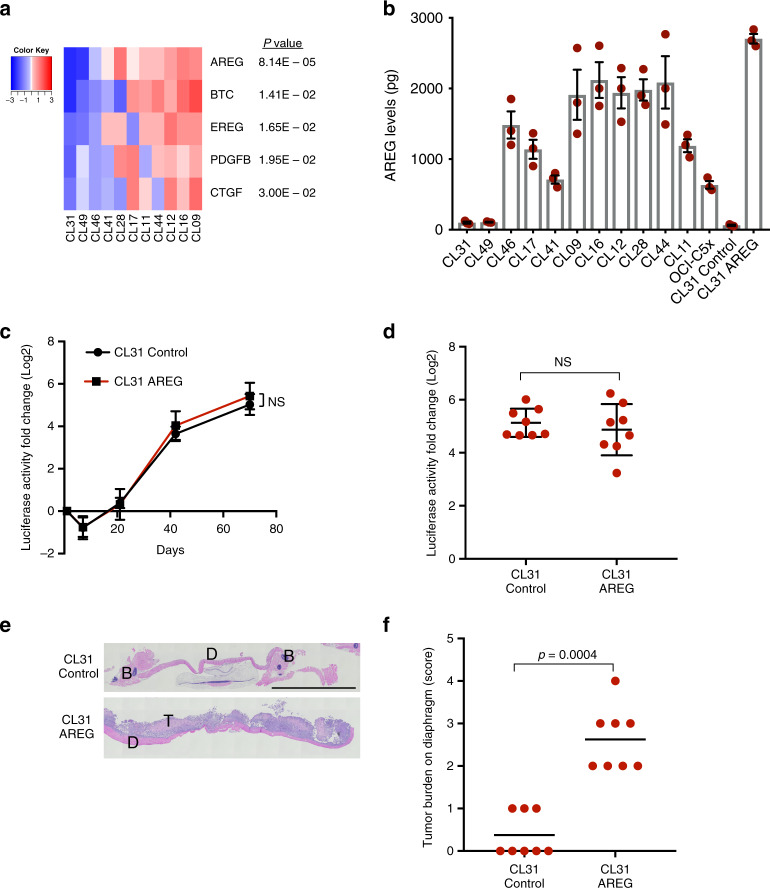
Overexpression of amphiregulin induces peritoneal metastasis of CL31. **a** Heatmap of ligands identified by RNA-sequencing. Growth factors and cytokines that were expressed by one or more of the other clones and poorly expressed in CL31 were identified and filtered for those whose corresponding receptor(s) were expressed in CL31 (cpm > 2). The relative mRNA levels of the identified ligands/growth factors are shown. Data are mean centered Log2. FDR-corrected *p* values were calculated using the exact binomial test in edgeR. **b** Levels of AREG secreted into the media by the indicated clonal populations over the course of 36 h, as determined by an ELISA assay. Data represent as mean ± SEM from three independent experiments. **c** Tumor burden in vivo assessed by measurement of luciferase activity in whole blood samples collected at the indicated time points. Data presented as fold change in luciferase activity compared to 24 h post injection. Representative of two independent experiments. Data summarized by mean ± SD, *p* values were computed using the Student’s *t* test and FDR corrected. **d** Fold change in luciferase activity in blood samples of individual mice collected at the end point (10 weeks) relative to the 24 h time point. The data shown as mean ± SD. *p* Values were computed using the one-way ANOVA test and Dunnett’s multiple comparison test. **e** Representative H&E images of solid peritoneal metastases on the diaphragm of mice inoculated with CL31 expressing AREG or vector control. T tumor cells, D diaphragm. Scale bar, 2 mm. **f** Tumor burden score of solid metastases on the diaphragms of mice inoculated with CL31 expressing AREG or vector control, determined as in Fig. 2e. *p* Value was computed using the chi-square test with Monte Carlo simulation. **d**–**f** Data from two independent experiments, total *n* = 8 mice.

We next investigated whether the ability of CL31 to form solid metastases in the clonal mixture is due to secreted factors that are supplied by one or more of the other clones. To identify such factors, we examined the expression of >470 ligand–receptor pairs from the database of ligand–receptor partners and identified 5 secreted factors that were lowest in CL31 (FDR *p* < 0.05) and whose corresponding receptor(s) were expressed in CL31 (Fig. 4a). Three of these factors were EGFR family ligands: amphiregulin (AREG), Betacellulin (BTC), and Epiregulin (EREG)] that activate ERBB2. AREG, in particular, was an attractive target because it was the most statistically significant factor that was distinguished in CL31 based on RNA-seq analysis. In addition, previous studies showed that AREG is present at elevated amounts in ascites fluid collected from advanced stage high grade serous ovarian cancer and lung cancer patients^32^, its expression has been associated with poor prognosis in several cancer types^33^, and it has been implicated in invasion of ovarian cancer cells^34,35^.

To test whether AREG is sufficient to induce solid metastases formation by CL31, we engineered this clone to exogenously express AREG at a level comparable to that observed in the other clonal populations (Fig. 4b and Supplementary Fig. 8A) and examined its aggressiveness and metastatic ability in two independent experiments. AREG overexpression in CL31 did not affect its tumor growth dynamics in vivo (based on Gluc activity in blood) (Fig. 4c, d), nor the cells doubling rate in vitro (Supplementary Fig. 8B). However, AREG overexpression in CL31 did induce the formation of solid peritoneal metastases on the diaphragm (Fig. 4e, f). This result indicates that AREG overexpression is sufficient to induce a phenotypic switch in CL31 from malignant ascites to solid metastatic phenotype.

### AREG acts during an early temporal window to induce solid peritoneal metastasis

**Fig. 5:**
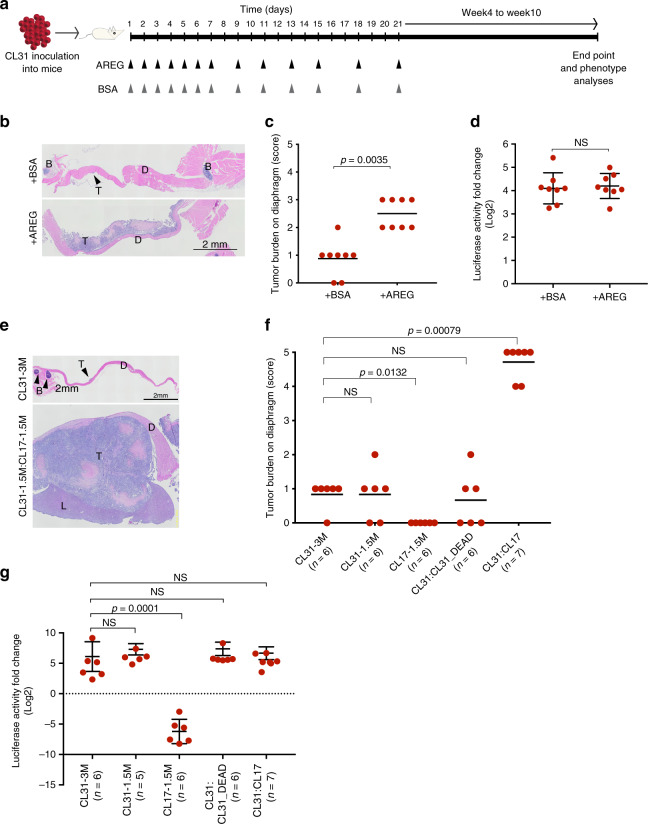
Transient exposure to CL17 or AREG is sufficient to promote peritoneal metastasis of CL31. **a** Schematic depiction of experimental design of short-term supplementation with human recombinant AREG in vivo. **b** Representative H&E images of diaphragms collected from mice at the 10-week end point supplemented with AREG or BSA vehicle control as depicted in (**a**), representative images from one of two independent experiments. T tumor cells, D diaphragm, B bone. Scale bar, 2 mm. **c** Tumor burden score of solid metastases on the diaphragm generated by CL31 in mice treated with AREG or BSA vehicle control. Data from two independent experiments, total *n* = 8 mice. *p* Value was computed using the chi-square test with Monte Carlo simulation. **d** Fold change in luciferase activity in blood samples collected from individual mice at the end point (10 weeks) relative to the 24-h time point. Data from two independent experiments (total *n* = 8 mice) and shown as mean ± SD. *p* Value was computed using the Mann–Whitney test, two-tailed. NS not significant. **e** Representative H&E images of the diaphragms of mice inoculated with CL31 alone (3 × 10^6^ cells) or a 1:1 mixture of CL31 and CL17 (3 × 10^6^ total cells). T tumor cells, D diaphragm, L liver, B bone. Scale bar, 2 mm. **f** Tumor burden score of solid metastases on the diaphragm generated by the indicated cell inocula. Data from two independent experiments (total *n* = 6–7 mice) and shown as mean ± SD. *p* Values were computed using the chi-square test with Monte Carlo simulation. NS not significant. **g** Fold change in luciferase activity in blood samples collected from individual mice at the end point (10 weeks) relative to the 24-h time point. Data from two independent experiments (total *n* = 6–7 mice) and shown as mean ± SD. *p* Values were computed using the one-way ANOVA test and Dunnett’s multiple comparison test. NS not significant.

The barcode analyses of the tumors generated from the multiclonal mixture as well as the growth dynamics of the individual clones (Fig. 1b, g) indicate that the representation of AREG-high clones is dramatically lower at 3- to 5-weeks after implantation in vivo, suggesting that if AREG contributes to the solid peritoneal metastasis of CL31 within the multiclonal mixture, it likely acts during an early temporal window to induce the phenotypic switch in CL31 and is later dispensable. To test this hypothesis, we examined whether brief supplementation with recombinant human AREG during the three weeks following injection of CL31 alone could induce the metastatic phenotype. In an attempt to mimic the gradual decrease of AREG provided by the clones in vivo (due to continuous cell death), mice were injected intraperitoneally with either recombinant human AREG (5 μg/mouse) or vehicle control over the course of the first 3 weeks as follows: daily administration starting on day 1 during the first week, then every other day in the following week, and twice a week during the third week (Fig. 5a). Supplementation with recombinant human AREG significantly increased the formation of solid peritoneal metastases by CL31 (Fig. 5b, c) compared to vehicle control without significantly affecting tumor cell growth rate (Fig. 5d). This data suggests that AREG availability during the initial and brief temporal window is sufficient to induce the phenotypic switch in CL31 to produce solid peritoneal metastasis and that this phenotype is not due to increased tumor growth in vivo. This data also supports our hypothesis that the transient presence of AREG-high non-tumorigenic clones may be sufficient to induce this phenotypic switch in CL31 in vivo.

### Transient and non-tumorigenic AREG-high clone promotes CL31 phenotypic switch

To further examine whether the transient presence of a non-tumorigenic AREG-high clone is sufficient to induce the phenotype switch in CL31, we assessed the phenotype of a pairwise combination of CL31 with CL17, an AREG-high non-tumorigenic clone. CL17 was a particularly ideal clone for this experiment since it is the only AREG-high clone that continuously decreases in cell number throughout the experimental time frame and its Gluc levels in blood reached background levels at around 5 weeks (Fig. 1b), suggesting that this clone is short-lived. We found that a 1:1 mixture of CL31 and CL17 (3 × 10^6^ total cells), but not CL31 alone (either 1.5 × 10^6^ or 3 × 10^6^ cells), generated large solid peritoneal metastases, most prominently on the diaphragm with extension to the liver (Fig. 5e). This result was not due to secondary effects induced by rapid death of CL17 (e.g., due to an inflammatory response), as a 1:1 mixture of live and dead CL31 cells (generated by repeated freeze–thaw) failed to recapitulate the metastatic phenotype of the pairwise CL31:CL17 mixture (Fig. 5f). Notably, the overall tumor burden (based on blood Gluc levels) of mice injected with CL31:CL17 was comparable to that of CL31 alone (Fig. 5g), indicating that the metastatic phenotype is not due to increased tumor cell growth. These results suggest that a transiently existing non-tumorigenic clone is sufficient to confer solid metastasis formation ability on CL31.

**Fig. 6:**
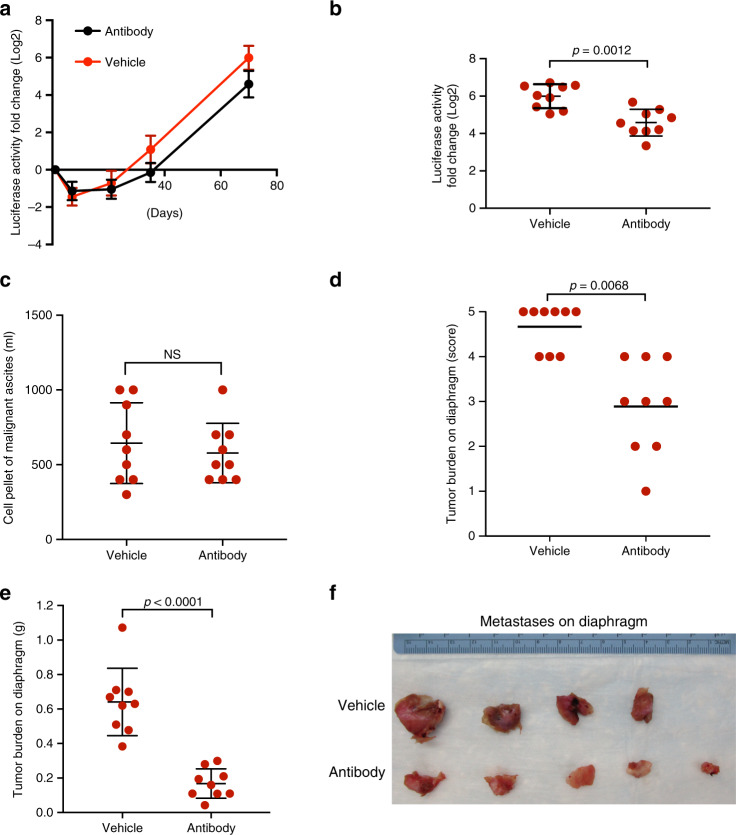
AREG blocking antibody reduces solid peritoneal metastasis. **a** Tumor growth dynamics of an equal mixture of CL31 and CL17 (CL31:CL17) treated with 200 μg monoclonal AREG blocking antibody or vehicle control, assessed by measurement of luciferase activity in whole blood samples collected at the indicated time points. Data presented as fold change in luciferase activity compared to 24 h post injection. Data represents mean ± SD from two independent experiments, *n* = 9 mice each group. **b** Fold change in luciferase activity per mouse injected with CL31:CL17 determined as in (**a**) at the 10-week end point. *p* Value was computed using the Mann–Whitney test, two-tailed. **c** Malignant ascites cell pellet volume at the 10-week end point. *p* Value was computed using the Mann–Whitney test, two-tailed. **d** Tumor burden score of solid metastases on the diaphragm at 10-week end point generated by CL31:CL17 in mice treated with AREG blocking antibody or vehicle control. *p* Value was computed using the chi-square test with Monte Carlo simulation. **e** Weight of solid tumors on the diaphragms at 10-week end point of same tumors in (**d**). Data from two independent experiments, *n* = 9 mice in total for each group, and shown as mean ± SD. *p* Values from Mann–Whitney test, two-tailed. NS not significant. **f** Individual diaphragms from mice in one of the two experiments at the 10-week end point.

Next, we addressed whether the ability of CL17 to induce the phenotypic switch in CL31 is dependent on AREG. Following intraperitoneal injection of the CL31:CL17 mixture, mice were treated twice a week with either an AREG blocking antibody or vehicle control, and tumor cell growth was measured over time by quantifying blood Gluc levels. We found that treatment with AREG antibody reduced the overall tumor burden (based on blood Gluc levels, *p* = 0.012) (Fig. 6a, b); however, there was no significant reduction in the cell pellet volume of malignant ascites (Fig. 6c), suggesting that AREG antibody did not reduce the growth of CL31 tumor cells in the ascites. In contrast, the burden of solid metastases on the diaphragm was significantly lower in the AREG antibody-treated group compared to vehicle control (Fig. 6d–f), suggesting that AREG blocking antibody specifically interferes with the formation of solid peritoneal metastases.

To test the abundance of CL31 and CL17 in the resultant tumors of CL31:CL17 mixture, barcode representation analysis was performed on malignant ascites and solid masses on the diaphragm at the 10-week time point. The results showed that CL17 barcode reads were below background levels (defined as the barcode reads for CL11, which was not present in the mixture) suggesting that CL17 does not co-exist with CL31 in the solid peritoneal metastases or malignant ascites at the end point (Supplementary Fig. 9).

Together, the results from the above experiments involving multiple approaches to evaluate the role of non-tumorigenic clones and AREG in regulating CL31 metastasis provide strong evidence that transient and non-tumorigenic clones cooperate with CL31 to affect tumor aggressiveness and that the interclonal cooperation is, at least in part, dependent on AREG.

### AREG enhances mesothelial clearance

**Fig. 7:**
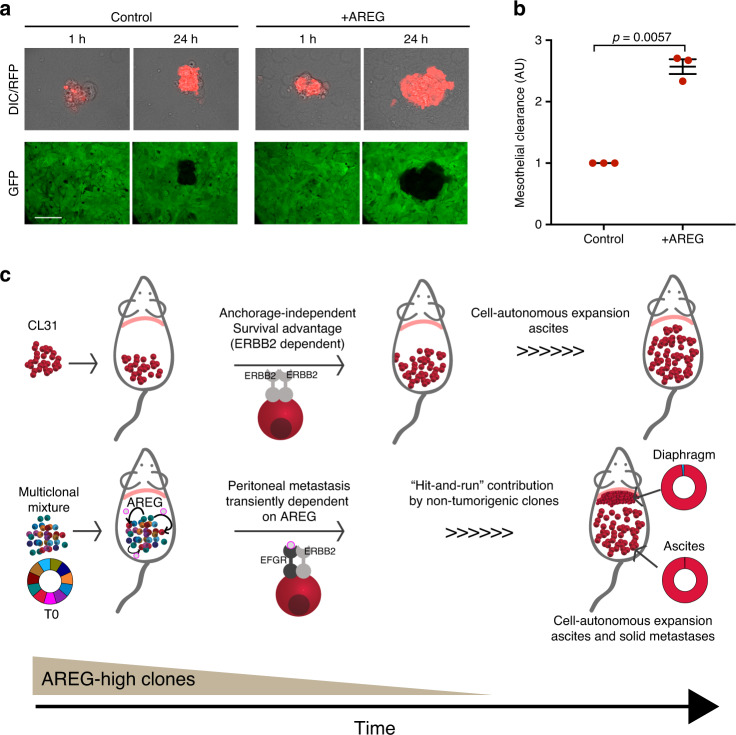
AREG enhances mesothelial clearance ability of CL31. **a** Representative differential interference contrast (DIC) and pseudocolored confocal fluorescence images of ability of vehicle- or AREG-treated CL31 cell clusters expressing red fluorescent protein (RFP) to clear a mesothelial monolayer expressing green fluorescent protein (GFP) at the indicated time points. Scale bar, 50 μm. **b** Quantification of the mesothelial clearance area (black area within the green monolayer) cleared in 24 h by CL31 spheroids treated with vehicle or AREG from three independent experiments. Mesothelial area cleared at the end point was normalized to the initial (1 h) area of CL31 clusters (measured from the DIC images), as previously described^39^. Relative clearance area of 20–30 clusters of CL31 of each condition per experiment were analyzed and averaged. Data shown as mean ± SEM of three independent experiments, shown in arbitrary units (AU), *p* value from Welch’s *t* test of the means. **c** Model illustrating the transient cooperative interactions involved in metastasis in this model system. CL31, which carries amplified *ERBB2* and displays anchorage-independent growth in vitro, forms only malignant ascites, but is unable to form solid peritoneal metastasis. Transient and non-tumorigenic AREG-high clones can act at an early temporal window, and are later dispensable, to induce CL31 solid peritoneal metastasis. AREG is required for metastasis of CL31, but not expansion in ascites or metastatic sites.

Dissemination of ovarian carcinoma cells to distant sites involves attachment to and clearance of the superficial layer of the mesothelium that encloses the organs in the peritoneal cavity^36–38^. To investigate whether the induction of CL31 metastatic ability by AREG is due to enhanced mesothelial clearance, we utilized a live-cell microscopy-based assay that we previously described^39^. Briefly, CL31 spheroids (from 100 cells each) were formed by overnight suspension culture, treated with either vehicle or recombinant human AREG (100 ng/ml) and clearance of a mesothelial monolayer was recorded over 24 h. We found that AREG treatment significantly enhanced the mesothelial clearance ability of CL31 (Fig. 7a, b). AREG treatment induced a similar enhancement of mesothelial clearance activity of CL49, an anchorage-independent clone with high expression of ERBB2 (Supplementary Fig. 10A, B). These findings suggest that AREG may induce the phenotypic switch in CL31 by enhancing mesothelial clearance.

### *ERBB2* amplification is enriched in solid peritoneal metastases of CCC patients

To address the relevance of these findings in human ovarian CCC, we evaluated whether *ERBB2* amplification is more prevalent in solid peritoneal metastases than primary ovarian tumors in a large cohort of unmatched CCC tumors. While paired samples from individual CCC patient tumors would provide a more meaningful assessment of enrichment of ERBB2 in peritoneal metastatic tumors, data on copy number variation in such pairs have not been reported and are not publicly available. *ERBB2* amplification was detected in only 1% of the primary site tumors compared to 7.00% of the solid peritoneal metastases (Fisher Exact test *p* = 0.027) (Table 1). This finding is consistent with a role for *ERBB2*-amplified cells in peritoneal metastasis and suggests that *ERBB2* amplification is acquired or selected for during the metastatic process.

**Table 1:** *ERBB2* amplification status in primary and metastatic samples of a CCC patient cohort.

	Amplified	Not amplified	Total	% Amplified
Primary/local	3	302	305	1.0
Peritoneal metastasis	3	40	43	7.0

Unmatched patient tumors from primary samples (*n* = 305) and peritoneal metastases (tissue: *n* = 43) were tested by NSG to determine *ERBB2* amplification validated using CISH. *p* Value from Fisher exact test.

## Discussion

In this study, clonal populations of tumor cells were used as a model system to examine functional heterogeneity and spatio-temporal nature of interclonal interactions. Systematic comparison of the phenotypic properties and clonal growth dynamics of individual clonal populations as well as clonal mixtures revealed the importance of transient interclonal crosstalk between a tumor-initiating clonal population and ostensibly innocuous non-tumorigenic clones to strongly influence metastatic behavior. The mechanism involved in this interaction was elucidated by identification of unique molecular features of the clonal populations as well as genetic and non-genetic perturbations to define the critical regulators of this crosstalk. These studies highlight not only the importance of transient interactions of neoplastic cells in tumor progression, but also the value of such experimental platforms using clonal populations of tumor cells in order detect transient functional interactions inaccessible to analyses in bulk tumor cell populations.

Clonal populations with heterogeneous metastatic phenotypes have been reported in the triple negative breast cancer cell line MDA-MB-231^2,3,40,41^. The findings from these studies show that each clonal population from this cell line expresses a set of intrinsic genes that determine its specific functional behavior, both with respect to metastatic ability, organ/tissue tropism, or responsiveness to the specific tissue environment^42^. Findings from our model system provide evidence for an alternate, non-cell autonomous mechanism for acquisition of metastatic activity and support the previous proposed hypothesis that clonal subpopulations within a tumor cooperate with one another to promote metastasis^11,13,43–46^. Importantly, our studies demonstrate that these cooperative interactions can be transient.

Ovarian tumor metastasis is believed to proceed through multiple steps, initially involving dissemination of tumor cells from the primary ovarian site into the peritoneum where there is selection for tumor cells that have acquired the ability to survive without cell–matrix interaction. Formation of solid peritoneal metastases requires additional steps, including intercalation into the mesothelial layer of peritoneal tissues and proliferation at those sites. The studies in this report are consistent with the model shown in Fig. 7c. Based on both the low frequency of *ERBB2* amplification in the original tumor and the OCI-C5x parental cell line, we propose that acquisition of *ERBB2* amplification is a late event in the primary ovarian-localized tumor, generating a small subpopulation of cells with elevated ERBB2 expression which strongly enhances anchorage independence, thus promoting survival and proliferation of tumor cells shed into the peritoneum. Soluble factors, such as AREG, secreted by the non-tumorigenic and transient clonal populations that act in a “hit-and-run” fashion and are later dispensable, induce the ability of the *ERBB2*-amplified tumor cells to initiate solid peritoneal metastasis by promoting mesothelial clearance. AREG is not critical for growth or survival of the *ERBB2*-amplied tumors cells, but rather strongly promotes metastasis. It is possible that in the absence of ligands, *ERBB2* amplification favors homodimerization of the ERBB2 receptors and thus maintains a ligand-independent and constitutively activated conformation that promotes anchorage-independent proliferation and survival^47^. When an EGFR associated ligand (e.g., AREG) is present, heterodimerization of EGFR-ERBB2 may promote prolonged and enhanced downstream signaling^48,49^ relative to that induced by ERBB2 homodimers.

Many studies have shown that AREG can promote invasion in multiple cancer models^34,50,51^. The mechanisms involved in AREG promotion of investion involve activation of ERK and PI3K pathways that regulate cell–cell adhesion, matrix metalloproteinase production, and mesenchyme transition^34,50,52^. Although AREG promoted solid metastasis of CL31, the solid tumor burden on the diaphragm generated by either AREG overexpression or treatment with exogenous AREG (Fig. 4e, f and Fig. 5b, c) was lower than that generated by the mix of all clones or the CL31:CL17 mixture, suggesting that additional secreted factors that act via EGFR–ERBB2 (e.g., EREG and BTC) or other receptor–ligand interactions also play a role in promoting solid metastasis in our model system.

The interclonal interactions leading to acquisition of metastatic capability could take place either in the primary tumor site prior to dissemination, where the non-tumorigenic clones are able to survive because they are supported by the microenvironment and are not challenged under anchorage-independent conditions in the ascites fluid, or within the peritoneum where small clusters of heterogeneous populations (*ERBB2*-amplified cells and AREG-high cells) are temporarily coexisting. We predict that within the peritoneum, the AREG-high cells transiently support invasion of *ERBB2*-amplified cells and later die or are outcompeted. The evidence that AREG-high clones do not co-exist with CL31 at later time points (neither in the ascites nor in the solid metastasis) support the requirement for only a transient interaction.

Our data demonstrate that AREG enhances mesothelial intercalation, providing one possible mechanism for the increased solid peritoneal metastases^53^. This hypothesis would be consistent with the transient requirement for AREG and the absence of clonal populations other than CL31 in the solid peritoneal metastases. However, given that AREG treatment of CL31 was not sufficient to fully recapitulate the extent of solid peritoneal metastases of the parental or multiclonal mixtures and AREG blocking antibody did not completely eliminate the solid peritoneal metastases burden, it is likely that other secreted factors or additional receptor–ligand pairs are involved in the clonal cooperation. This is further supported by the finding that knockdown of ERBB2 protein in CL31 only partially impaired its anchorage-independent growth, that only 18.9% on average of the cells comprising solid tumors generated by the parental line harbor *ERBB2* amplification, and that parental cells with low/negative ERBB2 expression were also capable of growing under anchorage-independent conditions, indicating that *ERBB2* amplification/expression is likely one mechanism among others that contribute to survival or metastasis in the parental line. It is likely that other clonal populations, which were not captured in our cloning process, are also able to metastasize.

There is evidence suggesting that coexisting heterogeneous tumor populations develop interdependencies since they support each other via symbiosis^7–9^. Our findings suggest the existence of commensal relationships, in which one population (CL31) benefits from another without benefit to the latter^43^. This implies that maintenance of tumor heterogeneity is not required for certain aspects of tumor progression, as temporal co-existence was sufficient to promote solid metastasis. Genetic analysis of the representation of clonal populations in primary versus secondary tumors has provided evidence that clonal populations are similar but not identical in primary and metastatic tumors^54^ and conversely, that rare subclones in the primary tumor can give rise to metastases^55,56^. McPherson et al.^57^ used mutational phylogeny analysis of autologous primary and distant peritoneal metastatic patient samples to show multiple modes of clonal spread in high grade serous ovarian carcinoma (HGSC). While a few samples showed a high degree of polyclonal mixing and reseeding of multiple clones at distant sites, the majority of the clonal diversity emerged at the primary site followed by monoclonal seeding to distant intraperitoneal sites. The “hit-and-run” commensal model we propose can provide one explanation for how this pattern of metastasis can occur and could also help explain the dearth of metastatic drivers (or biomarkers) identified to date. These findings are of clinical relevance as the subpopulations providing these prometastatic factors would no longer be required or detectable at the distant metastatic sites. Further, clones with a low mutant allele frequency could be critical to drive or support metastasis. Identification of this type of commensal cooperative mechanism would not have been feasible with bulk analysis at static time points and at specific site. This is also supported by our findings that *ERBB2*-amplified cells are rare in both the original patient sample (primary site) and in the parental cell line in vitro but are enriched for in the solid metastasis resultant from OCI-C5x inoculation in vivo. It is difficult to assess the generalizability of this mechanism of peritoneal metastasis in human CCC because static analyses of the clonal composition of matched primary and metastatic tumors fails to capture mechanisms involving unidirectional, transient interactions that drive metastasis but are dispensable for metastatic expansion. While ideally matched primary and metastatic samples, as well as longitudinal sampling would be ideal to support our finding’s relevance to patients, our analysis of primary and peritoneal metastasis (unmatched) of CCC samples are consistent with a role for *ERBB2*-amplified cells in peritoneal metastasis. In addition, a study by Brodsky et al. reported that amplification of *ERBB2* was evident in multiple HGSC metastatic samples and not in the matched primary tumor^58^, suggesting that *ERBB2* amplification is acquired or selected for during the metastatic process.

Interestingly, our findings are consistent with models from evolutionary biology and cooperation theory models which show that cooperation in groups is often transitory and that cooperators can decrease in frequency within groups over time^59,60^. In the model presented here, clusters that include ‘cooperative’ AREG-producing cells (e.g., CL17) may allow more effectively clearance of the mesothelium in order to initiate metastasis. The evidence that these “cooperative” cells could not be detected in metastases suggests that they are at a disadvantage and are evolutionarily outcompeted by CL31 cells within each cluster. Thus, ovarian cancer metastasis offers an intriguing model for studying the evolutionary dynamics of cooperation among cancer cells.

The use of this model system revealed a critical role of transient and seemingly harmless clonal populations in tumor metastasis and uncovered an additional dimension of the complexity of metastasis-driving mechanisms. While an understanding of the impact of intratumoral heterogeneity on tumor behavior is emerging, our findings demonstrate that spatial and temporal aspects of clonal interactions must be taken into consideration.

## Methods

### Generation of clonal populations and virus production

OCI-C5x cell line and the mesothelial cells (ZT-GFP) were a kind gift from Dr. Tan A. Ince^22^. ZT-GFP cells were cultured in a media of equal mix of 199 media (Gibco) and MCDB105 media (Sigma) (199:MCDB105 media) with 10% heat inactivated fetal bovine serum. OCI-C5x were cultured and passaged in OCMI-L full medium (United States Biological Cat# 506390) in a humidified incubator at 37 °C with 5% CO_2_. OCI-C5x was engineered to co-express tdTomato and and *Gaussia* Luciferase via lentiviral transduction, as described below. After transduction, single cells positive for tdTomato were sorted under sterile conditions into 384-well plates containing OCI-C5x-conditioned media (50 µl/well) using fluorescence-assisted cell sorting (FACSAria cell sorter; BD Biosciences, Inc.). OCI-C5x conditioned media was prepared by seeding 7.5 × 10^5^ OCI-C5x cells in a 10 cm plate with 10 ml OCMI-L media and collected 48 h after incubation. The media was spun down (3 min at 140 × *g*) and the supernatant was stored at −20 °C. Four hours after sorting, each well was visualized under a fluorescence microscope and only wells with one cell were included in our studies. Once cells reached confluence in the 384-well plate cells were trypsinized and replated into to larger wells with OCMI-L full media.

For barcode tagging of the clones, a lentivirus containing a unique barcode (Supplementary Table 1) was introduced to a specific clone in an infection efficiency of 30%, and then selected for infected cells by 1 μg puromycin for 4 days. Genomic DNA of each barcoded clonal population was isolated and equal amplification rate among all population was confirmed.

### Plasmids, shRNAs, and virus production

CSCW-GLuc-IRES-CFP was a generous gift from Dr. Bakhos Tannous lab^23^. To replace the CFP with tdTomato, we transferred the Kpn1-digested fragment from pBS-IRES-tdTomato-WRE plasmid into the Kpn1-digested CSCW-GLuc-IRES-CFP plasmid followed by standard quick ligation protocol. Overexpression and knockdown of specific target genes in these cells was performed via lentiviral transduction using standard protocols, following selection with 1 μg/ml Puromycin for 4 days. Flag-HA-AREG and Flag-HA-GFP were a generous gift of the Harper W. lab (Harvard Medical School, Boston, MA). shRNA vectors for ERBB2 were purchased from Dharmacon (RHS3979-201785743, RHS3979-201768731, RHS3979-201768734, and RHS3979-201768735). shRNA-GFP control (Addgene, #30323).

### Analysis of barcode distributions

The in vitro multiclonal mixture cultures were sampled each time they were passaged, i.e., approximately 2 × 10^6^ cells were collected following cell counting by centrifugation, and stored at −80 °C. Tumor tissue samples were also stored at −80 °C prior to genomic DNA isolation. Tumors were mechanically homogenized on ice and entire tumor tissue was transferred to an Eppendorf tube for genomic DNA isolation. The genomic DNA was isolated using the QIAamp DNA Mini Kit (Qiagen, Cat. No. 51306) and DNA concentrations were measured on the Epoch microplate spectrophotometer (BioTek). The barcodes were subsequently isolated by PCR on 3 µg genomic DNA per sample (maximum 1 µg per PCR) using a common forward primer and set of reverse primers with unique index sequences that allow for multiplexing of samples. Per sample, PCR products were combined and isolated using the QiaQuick PCR purification kit (Qiagen, Cat. No. 28106). The quality and concentration of the PCR products were determined using a 2200 TapeStation and D1000 screen tapes (Agilent Technologies). To generate the NGS libraries, samples were mixed in equal proportions. The libraries were isolated from a 2% agarose gel using the QiaQuick gel extraction kit (Qiagen, Cat. No. 28706) and their quality and concentration were determined using a 2200 TapeStation and D1000 screen tapes (Agilent Technologies), and q-PCR. Each library was sequenced on a MiSeq (Illumina) by the Biopolymers Facility at Harvard Medical School using the primers WSL_NGS_Barcode_Seq and WSL_NGS_Index_Seq (Supplementary Table 2).

Index sequences were used to demultiplex the samples. Barcode sequences that matched the 15xWS design, a Phred quality score of 10 or greater for each position and an average Phred quality score greater than 30, were selected for further analysis. First, we selected the barcode sequences that were used in the experiment and then we normalized the barcode counts to their mean fractions in the *T* = 0 reference samples. For each sample, the barcode fractions were computed by dividing the number of reads of a given barcode by the total number of reads for all barcodes in that sample (Supplementary Data 1, Raw barcode counts).

### Doubling time

Each population was plated into a 6-well multiwell plate (Corning) at a density of 7 × 10^3^ cells. Cells were harvested by trypsinization on days 1, 3, and 5 and counted using a particle counter (Z1; Beckman Coulter, Inc.). Experiments were carried out in triplicate. Doubling time was calculated with the assumption of exponential growth. The number of generations was calculated using the following formula: [((Day 1) − (Day 5))/l(2)]. Doubling time was derived by dividing the duration of the experiment (96 h) by the number of generations.

### Soft agar assay

The soft agar assay was performed in 6-well plates, the assay was carried out in duplicates for each tested group. For each tested group 2 × 10^4^ cells were added to 1 ml of 0.4% low-melt agarose solution (Sigma) in OCMI-L media and transferred to a well with a bed of growth media with 0.5% low-melt agarose. At day 21, viable colonies were stained with iodonitrotrazolium chloride (25 mg/ml) (Sigma-Aldrich). Subsequently colonies were imaged with a dissecting microscope (2.5×). ImageJ was utilized to count and analyze particles. Data were analyzed using GraphPad Prism (GraphPad, Inc.). Images shown are representative of at least three independent experiments.

### Copy number analysis

Genomic DNA was extracted from each sample using the DNeasy Blood & Tissue Kit (Qiagen, 69504), followed by acoustic sonication using Covaris S220 sonicator. DNA concentration was quantified using the BioTek microplate reader (BioTek Instruments, Inc.). Next, the DNA was sparse-sequenced to infer the copy number profiles. The sequencing libraries were constructed by adding 3′ adenylation and ligating barcoded sequencing adaptors to the fragmented DNA using NEBNext DNA Library Prep Master Mix set for Illumina (NEB, E5040L). The generation of barcoded sequencing adapters can be found in ref. ^61^. The barcoded libraries were pooled and sequenced on a sequencing lane of Illumina HiSeq 2500. The sequencing data was analyzed following the informatics procedure in Baslan et al.^62^. Briefly, the sequencing reads were mapped to the human assembly hg19 using bowtie 2 and the Genome Analysis Toolkit (GATK) was used to locally realign the BAM files at interval that had INDEL mismatches before PCR duplicate marking with Picard. Copy number was calculated from read density by dividing the genome into variable bins and counting the number of unique reads in each 200 kb interval. Bins at the centromeric and telomeric regions were filtered to remove false-positive errors. We then applied Loess normalization to correct for GC bias^62^. The copy number profiles were segmented using circular binary segmentation as in Olshen et al.^63^.

### Whole-exome sequencing and mutation calling

Whole-exome libraries were prepared from DNA extracted from the individual clones, parental line, and the patient’s blood. DNA was sonicated to 150 bp using the Covaris e220. Libraries were created using either the Agilent SureSelect XT protocol, or the Kapa HyperPrep protocol (Kapa Biosystems). Hybrid capture was conducted for 24 h using the Agilent human all exon v4 + UTR capture baits. For two samples (patient blood and parental line) an additional library was created using the Kapa HyperPrep protocol and captured using the Agilent human all exon v5 baits. Libraries were 100 bp paired-end sequenced on a Illumina HiSeq 2000.

Reads were aligned to hg19 using BWA mem, local realignment and quality score recalibration were conducted using GATK, PCR duplicates were marked using Picard Tools. Somatic mutation calls were made using Mutect (v 1.1.7), patient blood was used as the match normal, calls were filtered against the dbSNP database (v 137) and rescued if they were present in the COSMIC (v 54) databases. As a final filter, mutation calls were required to have 30× depth in the tumor, 10× in the normal, at least a 10% mutant allele frequency in the tumor and less than 2% in the normal. After the full list of filter-passing mutations was generated, samtools mpileup was generated for each mutation site in each of the clones to look for the presence of the mutant reads below our initial calling threshold. Functional annotation of mutation calls were conducted using Oncotator (http://portals.broadinstitute.org/oncotator/).

Mutational signature analysis was conducted using a list of all filter-passing mutations for each sample (Supplementary Data 2, all mutants tab). The COSMIC trinucleotide signatures were used for cosine similarity signature assignment (https://cancer.sanger.ac.uk/cosmic/signatures/SBS) using the mutational patterns R package (https://github.com/UMCUGenetics/MutationalPatterns). Signatures were filtered to only include those contributing at least 200 total mutations across all samples.

### Tree building

Whole-exome mutations were filtered to include only those present at 30× depth across all samples, and present at a VAF ≥ 0.25 in at least one sample. The 7,402 resulting mutations were the classified as present in a sample (VAF ≥ 0.25), absent (VAF < 0.10), or ambiguous (0.10 ≤ VAF < 0.25). The resulting matrix was then input into mpboot to generate a maximum parsimony tree using 1000 bootstrap iterations^64^. All nodes had bootstrap support of 1.0. For the mutations composing each branch, the mean VAF of those mutations in the parental clone was determined, and the branch colored accordingly.

### RNA sequencing

Total RNA was extracted from each sample using the RNeasy Mini Kit according to manufacturer’s protocol (Qiagen, Inc.). The RNA concentration was measured using the Epoch microplate spectrophotometer (BioTek). Prior to library generation for RNA-sequencing, the quality of the RNA was determined on a 2200 TapeStation analyzer using RNA screen tapes (Agilent Technologies). The mRNA libraries were generated by the Biopolymers Facility at Harvard Medical School (http://genome.med.harvard.edu, Boston, MA) and included poly-A enrichment and the Directional RNASeq Wafergen services. The quality of these libraries was assessed on a 2200 TapeStation analyzer using D1000 screen tapes (Agilent Technologies), and by qPCR. In total, 12 mRNA libraries (one sample for the OCI-C5x parental cell line, and one sample of each clonal population) were multiplexed into one pool and sequenced on Illumina HiSeq2000 instrument (Illumina Inc., San Diego, CA). Adapter sequence was trimmed using Trimmomatic-0.30. Trimmed reads were mapped with bowtie2-2.1.0 to the hg19 version of the human genome. The HTSeq-0.5.4 and EdgeR packages were used to quantify counts per gene and perform differential expression analysis, respectively.

Growth factors/cytokines and associated receptors were identified from AmiGO 2 (http://amigo.geneontology.org/amigo) and the Database of Interacting Proteins (https://dip.doe-mbi.ucla.edu/dip/DLRP.cgi). Hierarchical clustering was performed in R 3.5.1 using Spearman correlation and average linkage. Heatmaps were generated with the heatmap.2 package. RNA-seq data has been submitted to the Gene Expression Omnibus (GEO), GSE123426.

### RPPA analysis

A vial of cells from each of the lines was thawed and plated on 10-cm cell culture dishes. Cells were collected by washing with ice-cold phosphate-buffered saline (PBS), scrapping, and centrifugation at 900*g* at 4 °C for 10 min. Pellets were resuspended in RPPA lysis buffer (1% Triton X-100, 50 mM HEPES pH 7.4, 150 mM NaCl, 1.5 mM MgCl_2_, 1 mM EGTA, 100 mM NaF, 10 mM NaPPi, 10% glycerol, 1 mM Na_3_VO_4_, and protease inhibitor [Roche]) and incubated on ice with occasional shaking for 20 min. The lysates were centrifuged at 13,000*g*, 4 °C, for 10 min. Lysed were denatured by adding 1% sodiumdodecyl sulfate (SDS) and boiling for 5 min. Each sample was diluted in five 2-fold serial dilutions and printed onto nitrocellulose- coated glass slides (Grace Biolabs) with an automated robotic Aushon arrayer (Aushon Biosystems). Each slide was probed with a validated primary and secondary antibody. Signal intensity was measured by scanning the slides with ImageQuant (Molecular Dynamics) and quantified using the MicroVigene automated RPPA module (VigeneTech Inc.). Relative protein levels were then determined for each sample. Signal intensity data were collected and analyzed using software developed specifically for RPPA analyses (http://www.VigeneTech.com). Linear intensity data were subjected to unpaired Student’s *t* tests in Rv.3.5.1. Significant antibody probes were defined as *p* < 0.05. Heatmaps represent the average of triplicate samples (log2-transformed and median-centered).

### Animals and in vivo procedures

*Xenograft studies*: All animal studies were performed according to protocols approved by the Harvard University’s Institutional Animal Care and Use Committee (IACUC). Eight-week-old female NOD-*scid* IL2Rgamma^null^ (NSG) mice were purchased from the Jackson Laboratory (Bar Harbor, Maine). For each experiment, Gluc-tdTomato expressing cells were injected intraperitoneally to 8–10 weeks old female NSG mice. The mice were euthanized 10 weeks after tumor cell injection. Each mouse was dissected and visually inspected for solid tumor metastasis under a fluorescence dissecting microscope for tdTomato signal, organs with tumors were collected, fixed with 10% formalin (Westnet-Simport), paraffin embedded and sectioned for H&E staining. Peritoneal cavity was washed with 1× PBS to collect malignant ascites into graduated 15 ml conical tubes, then the cells were spun down and the volume of cellular pellet was assessed by the volume marks on the tube since precise measurement of the pellet volume is difficult due to its viscosity. An aliquot of the malignant ascites for each sample was mixed with equal volume of histogel (VWR cat# 83009-992), incubated for 20 min in room temperature to solidify, fixed in 10% formalin followed by paraffin embedding, sectioning and H&E staining. Three to five mice per group were used for each experiment and at least two independent experiments were performed.

*Solid metastases tumor burden scoring*: On completion of immunohistochemical staining of the tissue samples, a pathologist examined the tissue slides in a blinded manner and documented the tumor burden. The classifications of tumor burden were based on a five-point scale: 0, no tumor cells; 1, few tumor cells; 2, few and small clusters of tumor cells; 3, large clusters of tumor cells, deposited along large area of the diaphragm; 4 and 5, bulky tumor deposits on part or the entire diaphragm, categorized as either 4 or 5 depending on the size of the deposits within this group. Two slides were scored for each sample and the average of the score was calculated.

*Solid metastases tumor burden analysis by weight*: Tumor masses identified on the diaphragm were cut out from the diaphragm and weighed. Tumor weight, in addition to the tumor burden scoring method, was used to monitor effect of amphiregulin blocking antibody on tumor burden since in this specific experimental design both the control and the tested groups had weighable tumors for comparison.

*In vitro Gaussia luciferase (G-Luc) activity*: Blood from the submandibular vein of each mouse was collected into tubes with Heparin (Henry Schein animal health supply, Ohio) at 24 h after tumor cell injection and every week thereafter for up to 10 weeks. Five microliters of blood from untreated mice (negative control) and of treated mice were transferred into each well of white 96-well plate in duplicate. To test the G-Luc activity in blood, coelentrazine solution (Prolume LTD, AZ) at a final concentration of 20 μM was prepared with DMEM media (Life technologies) and incubated for 30 min at room temperature, then 50 μl of the coelentrazine was added using multichannel pipettor to each well and bioluminescence signal was measured immediately using a plate luminometer (MLX luminometer, Dynex Technologies, Chantilly, VA). Fold change in G-Luc activity was calculated by normalizing to Day 1 G-Luc levels (24 h after injection). G-Luc fold change for each mouse was averaged for each time point and graphed on a log scale using GraphPad Prism.

*Amphiregulin blocking antibody in vivo*: A total of 3 × 10^6^ cells of an equal mixture of CL31 and CL17 were injected I.P. to 8–10-week-old female NSG mice. Immediately after cell injection, mice were treated with 200 μg monoclonal AREG blocking antibody (AREG 37.4 Sigma-Aldrich, Israel) in a 200 μl PBS (I.P. injection), twice a week for 10 weeks. Control group was treated with PBS vehicle control. Tumor growth dynamics was monitored by measuring G-luc activity from blood and tissues were collected as mentioned above. Solid masses on the diaphragm were collected and weighed.

*Human recombinant AREG peptide in vivo*: A total of 3 × 10^6^ cells were injected I.P. into 8–10-week-old female NSG mice. Immediately after cell injection, mice were treated with human recombinant Amphiregulin (Cat# 100-55B, Peprotech) dissolved in 0.1% bovine serum albumin (BSA) (Sigma) to a final concentration of 1 μg/μl. Mice were treated by I.P. injection with either 5ug/mouse AREG in 200 μl sterile water (Gibco) or BSA vehicle control (0.0025% BSA final concentration) as follows: starting on the day of cell inoculation and every day for a week, then every other day on the second week and twice a week on the third week. No AREG supplementation from week 4 through week 10.

### FISH analysis

Tumor blocks of FFPE were sectioned (5 µm in thickness). For each specimen, one sectioned slide was stained with H&E and used by rodent pathologists to mark tumor areas. For the original patient tumor sample, the tumor area was identified by pathologist and by S100A1 staining that differentiates carcinoma cells from normal epithelial cells in the ovary. The sections were delivered to the Cytogenomics Core laboratory (cytogenomics.bwh.harvard.edu) at the Brigham and Women’s Hospital (Boston, MA) for HER2 testing following manufacturer protocol of a commercial two-color FISH probe set (PathVysion, Abbott Molecular). For each sample 60–100 cells were scored for ERBB2:CEP17 ratio. Each sample was scored independently by two technicians.

### **Immunohistochemistry staining**

Formalin-fixed tumor samples derived from mouse xenografts were processed and embedded in paraffin. Tissue sections were deparaffinized and antigen retrieval was achieved by use of heat-induced epitope retrieval with pH 6.0 citrate buffer (Sigma). Tissue sections were stained with H&E, anti-ERBB2 antibody (Dako, A0485, dilution 1:200), or anti-S100A1 antibody (Dako, Z031129-2, dilution 1:00). Immunostained slides were counterstained with hematoxylin (Sigma). Images of tissue sections were captured at 10× magnification to create a merged image of the entire section using Olympus VS120 Slide Scanner.

### Secreted amphiregulin measurement

OCI-C5x parental cells and each individual clone were seeded at the same density (7.5 × 10^5^ cells in a 10 cm plate) with 5 ml of 199:MCDB105 media with 2% heat inactivated fetal bovine serum and incubated for 48 h. The media was collected and spun down (3 min at 140 × *g*) and the supernatant was used or alternatively, kept at −20 °C. ELISA test for AREG levels was performed using 100 µl of each sample in duplicates and following the manufacturer protocol (Abcam, Ab99975).

### Mesothelial clearance assay

Comparable size multicellular spheroids of CL31 were prepared by culturing 100 cells of CL31 per well on low attachment 96-well round bottom plates (Westnet, 7007), cells were cultured in a media of equal mix of 199 media (Gibco) and MCDB105 media (Sigma) (199:MCDB105 media) with 2% fetal bovine serum and incubated overnight at 37 °C to promote spheroid formation. Concurrently, 250,000 ZT mesothelial cells per well were plated on fibronectin-coated (5 μg/ml, Sigma-Aldrich) 24-well glass-bottom culture dishes (MatTek corporation, P24G-1.5-13-F). The mesothelial cells were incubated overnight at 37 °C to form confluent monolayers. After the 24-h incubations, 10–15 multicellular spheroids of each condition were transferred to the wells containing the mesothelial monolayers in duplicates wells. The spheres were allowed to settle for two hours prior to starting live imaging. Imaging was performed using a Nikon Spinning disk confocal microscope with the integrated Perfect Focus System and low (×20, 0.75 NA) magnification/NA DIC optics, Nikon fast (<100-ms switching time) excitation and emission filter wheels, Nikon linear-encoded motorized stage, Hamamatsu ORCA-AG cooled CCD camera, custom-built microscope incubation chamber with temperature and CO_2_ control, Nikon NIS Elements AR software v3, and TMC vibration-isolation table. Over 20 spheroids were imaged per condition. Phase-contrast, GFP and RFP images were captured every 20 min for 24 h. To quantify the mesothelial clearance area, the non-fluorescent area, created by the invading spheroid, in the GFP mesothelial monolayer images was measured at 24 h and divided by the initial area of the cancer spheroid at time 0. All measurements were taken using ImageJ software.

### **Quantitative PCR**

mRNA prepared from cell extracts using the RNeasy Mini Kit (QIAGEN) was reverse-transcribed into cDNA using the qScript cDNA synthesis kit (Quanta Biosciences). Real-time PCR was performed on an ABI PRISM 7900HT or QuantStudio 7 Flex Real-Time PCR System (Life Technologies) with Power SYBR Green PCR Mix (Life Technologies). Amphiregulin and RPLPO primers sequences as indicated in Supplementary Table 2.

### Immunoblot assay

Samples were lysed in cell RIPA lysis buffer (Boston Bioproducts) supplemented with protease and phosphatase inhibitor cocktails (Roche life science). Protein concentrations were determined by BCA protein assay (Life Technologies). Equal protein amounts were denatured in SDS buffer (1% SDS, 8% Glycerol, 62.5 mM Tris-HCL, pH 6.8) with 1.5% β-mercaptoethanol for 5 min. Protein were analyzed by sodium dodecyl sulfate polyacrylamide gel electrophoresis and immunoblot using indicated antibody [Anti-ERBB2 Thermo Fisher Scientific MS325-P1, (dilution:1:500), Anti BCL-X_L_ (dilution:1:500), Cell Signaling 2764 S, β-Actin, Cell Signaling 3700S (dilution 1:500)]. Blots were imaged with either Odyssey CLx infrared imaging system (LI-COR) or Kodak film developer. Western blots were scanned using an Epson 3000 scanner and are representative of at least two independent experiments. (see Supplementary Fig. 11 and Supplementary Fig. 12 for full scans of blots presented in this study).

### Flow cytometry

Cells were cultured in OCMI medium and harvested with Trypsin–EDTA during log phase growth. Cells were washed twice with 1× PBS and blocked with 5% goat serum in PBS. For ERBB2 stain, anti-ERBB2 primary antibody pre-conjugated with secondary antibody (Brilliant Violet) (Biolegend, 324420) was used. Cells were sorted and analyzed at the Dana-Farber Flow Cytometry Core Facility (flowcytometry.danafarber.org) on a FACSCalibur (BD Biosciences, Inc.). Experiments were carried out in triplicate. Flow cytometry data was analyzed using FlowJo v7.6.5 (FlowJo LLC) and GraphPad Prism (GraphPad Software, Inc.).

### Analysis of *ERBB2* amplification status in human ovarian CCC

Clear cell ovarian tumors were tested with comprehensive tumor profiling including NGS using the NextSeq platform (Illumina, Inc., San Diego, CA). A custom-designed SureSelect XT assay was used to enrich 592 whole-gene targets (Agilent Technologies, Santa Clara, CA). The CNA of each exon is determined by calculating the average depth of the sample along with the sequencing depth of each exon and comparing this calculated result to a pre-calibrated value. Copy number of 6 or higher was called amplified. Chromogenic in situ hybridization was used for confirmatory testing (INFORM HER-2 Dual ISH DNA Probe Cocktail); HER2 amplification was defined as Her2/chr17 ratio ≥2.0. This study was conducted in accordance with guidelines of the Declaration of Helsinki, Belmont report, and U.S. Common rule. In keeping with 45 CFR 46.101(b)(4), this study was performed utilizing retrospective, deidentified clinical data. Therefore, this study is considered IRB exempt and no patient consent was necessary from the subject.

### Statistics

The specific statistical tests used and resulting *p* values are indicated in the figure legends. Unless indicated otherwise, all statistical tests were carried out using Graphpad Prism version 7.0. Statistical analysis for the tumor burden scoring was performed using R software.

### Reporting summary

Further information on research design is available in the Nature Research Reporting Summary linked to this article.

## Supplementary information

Supplementary Information

Description of Additional Supplementary Files

Supplementary Data 1

Supplementary Data 2

Reporting Summary

## Data Availability

The RNAseq data have been deposited in the Gene Expression Omnibus (GEO), GSE123426. The raw FASTQ files of the copy number data have been deposited into the Sequence Read Archive (SRA), accession ID: PRJNA658185. The raw data of the Whole-Exome Sequences have been deposited into Sequence Read Archive (SRA), accession ID: PRJNA668770. All the other data supporting the findings of this study are available within the article and its supplementary information files and from the corresponding author upon reasonable request. A reporting summary for this article is available as a Supplementary Information file.
