# Supplementary material for: Transient commensal clonal interactions can drive tumor metastasis
Source: Nat Commun. 2020 Nov 16;11:5799. doi: 10.1038/s41467-020-19584-1 (PMC7669858; doi:10.1038/s41467-020-19584-1)
Supplement: Supplementary file 1 — Supplementary Information [file 41467_2020_19584_MOESM1_ESM.pdf]

Supplementary Information

Transient Commensal Clonal Interactions Can Drive Tumor Metastasis

Naffar-Abu Amara *et al.*

Supplementary Figure 1.

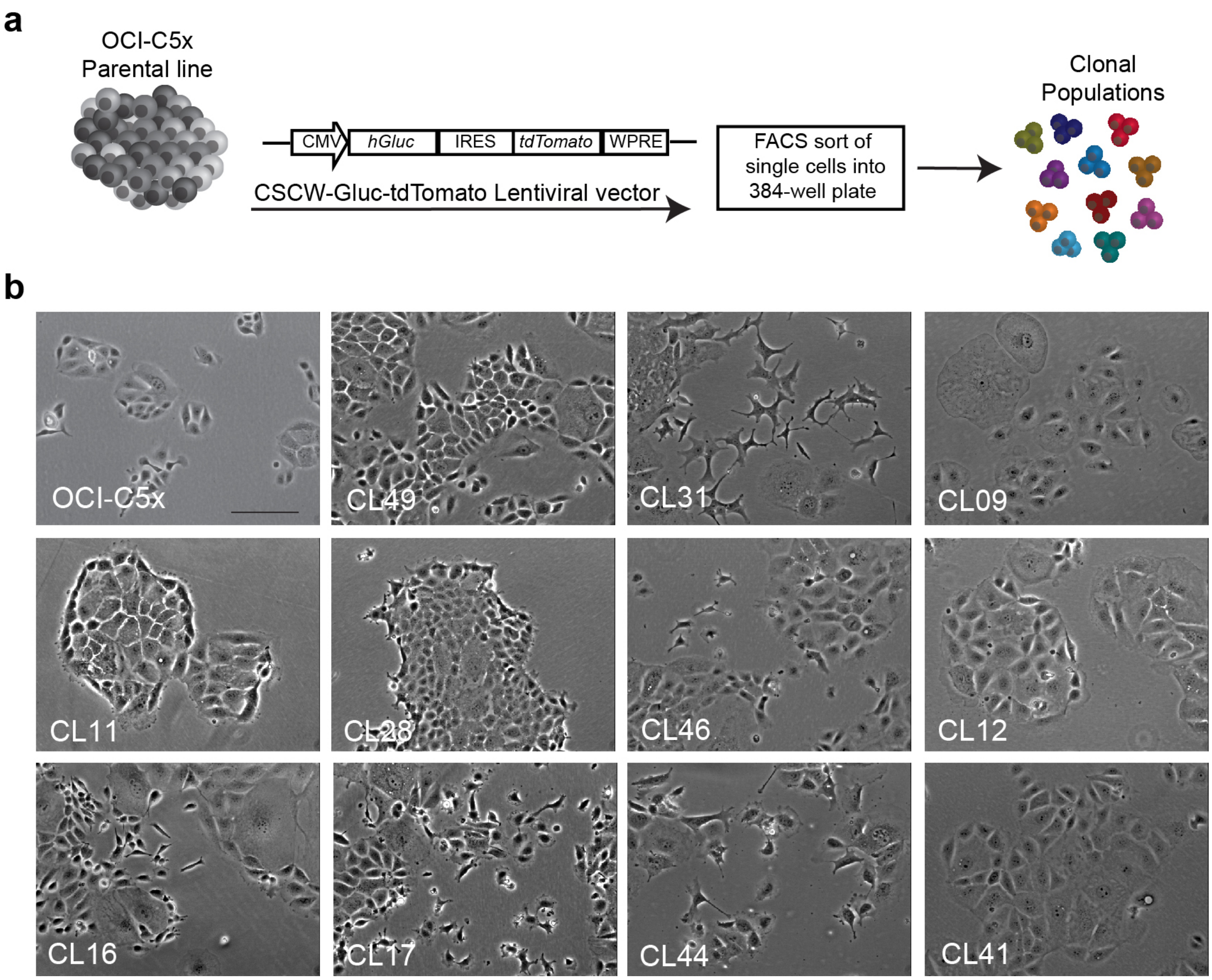

Supplementary Figure 1: Clonal populations derived from OCI-C5x cell line vary in morphology.

(A) Schematic description of generation of the clonal populations from OCI-C5x. (B) Representative phase contrast images of parental OCI-C5x line and clonal populations, representative images from one of more than three independent experiments. Scale bar, 100μm.

Supplementary Figure 2.

a

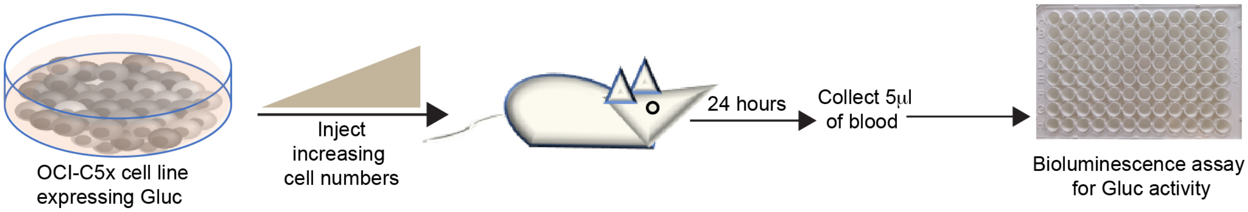

b

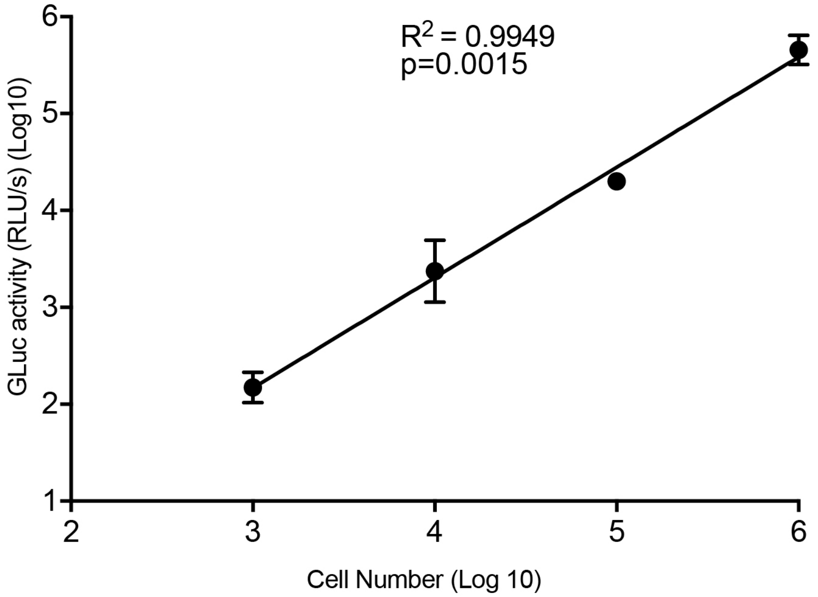

Supplementary Figure 2: Measurement of tumor growth dynamics by luciferase assay.

(A) Increasing number of OCI-C5x cells expressing Gaussia luciferase (Gluc) were injected intraperitoneally into NSG mice. Blood samples were collected 24 hours post injection and Gluc activity was measured on 5μl of blood in duplicates. Values are the average of the duplicates. (B) Linear regression of number of injected cells with *ex-vivo* luciferase activity in blood detected at 24 hours post injection. Each point represents the mean ± SD of Gluc values from two independent experiments, n=7 mice. *p* =0.0015, two-tailed t test.

Supplementary Figure 3.

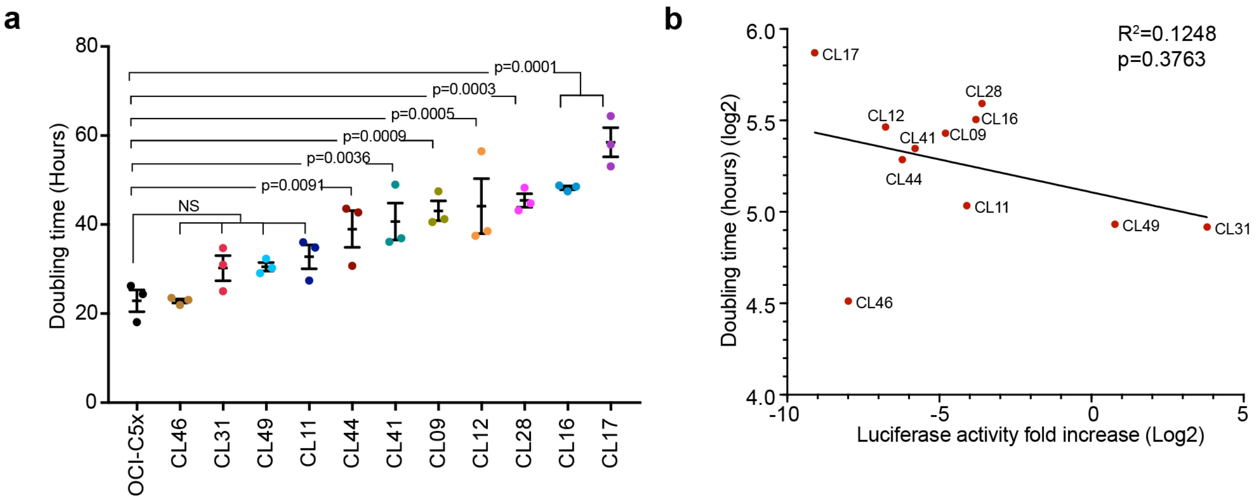

Supplementary Figure 3: Clonal populations derived from OCI-C5x cell line vary in population doubling time.

(A) Doubling time of OCI-C5x and clonal lines grown in triplicate and for over 5 days. Doubling time was computed as follows: 24*[(LOG (cell number day 1)-LOG (cell number day 5))/LOG (2)]. Data shown as mean± SEM from three independent experiments. *p* values from one-way ANOVA, corrected for multiple comparisons using Dunnett's method. (B) Linear regression analysis of doubling time and tumor burden (measured by luciferase activity in blood samples collected at 10 week end point) for all 11 clonal populations. *p* =0.3763, two-tailed.

Supplementary Figure 4.

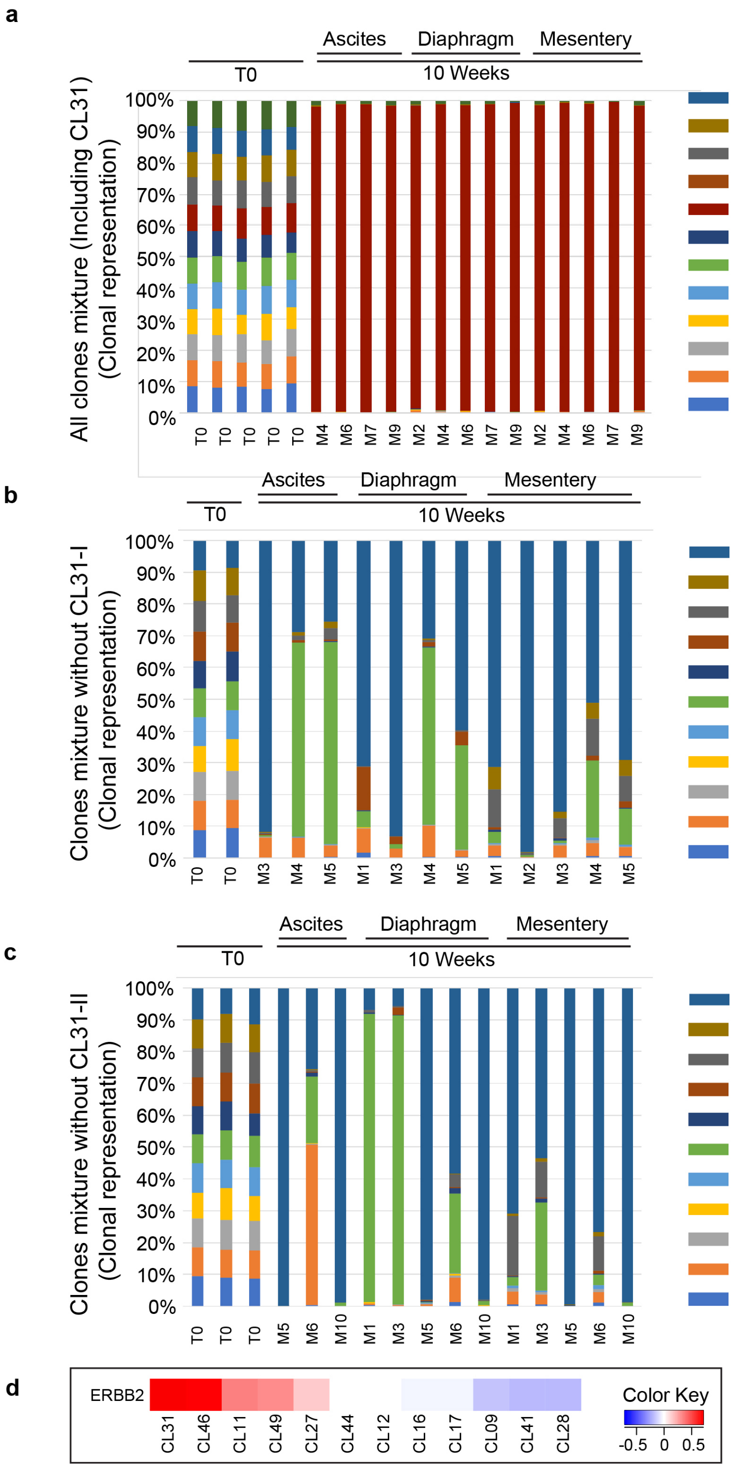

Supplementary Figure 4: CL49 dominates the multiclonal mixture when excluding CL31

(A) Barcode representation in the indicated samples collected from mice injected with the multiclonal mixture composed of all barcoded clones, and (B and C) in samples collected from multiclonal mixture excluding CL31 of two independent experiments. (D) RPPA analysis of ERBB2 expression in clonal populations. Heatmap shows the average ERBB2 expression of triplicate samples (log2-transformed and median-centered).

Supplementary Figure 5.

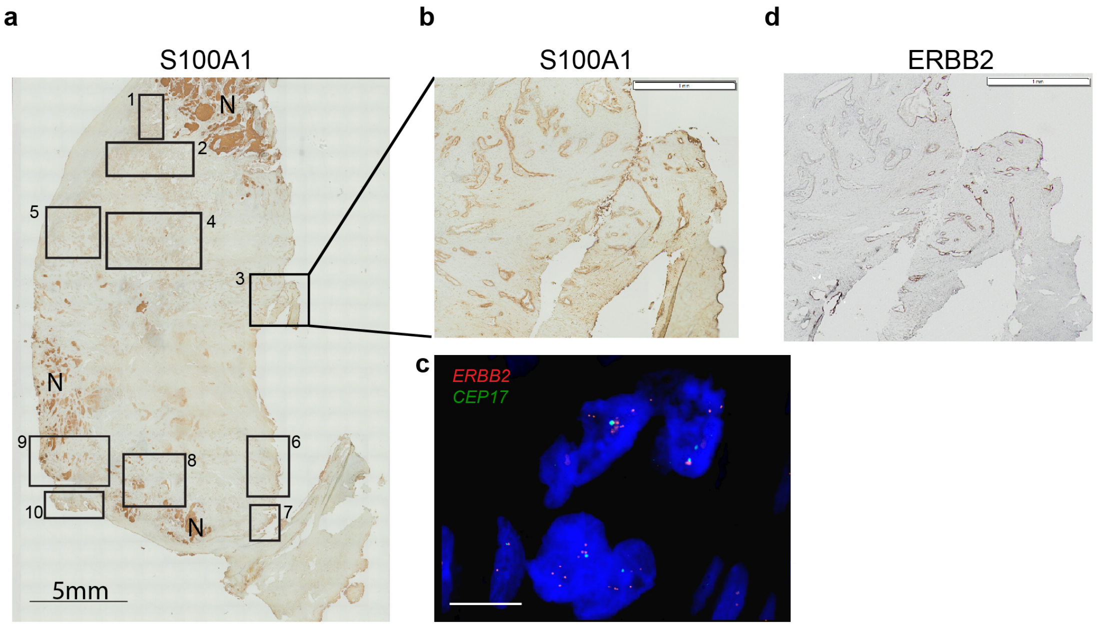

e

Region # / *ERBB2*:CEP17 score	1	2	3	4	5	6	7	8	9	10
% of scored cells with *ERBB2*:CEP17≥2	0	1.66	26	23.3	13.3	25	16	10	20	0
Average of *ERBB2*:CEP17 ratio from cells above	NA	2	2.96	2.42	2.6	2.7	2.46	2	2.5	NA

Supplementary Figure 5: Identification of *ERBB2*-amplified cells in the original patient sample from which OCI-C5x was derived.

(A) Histology section of the patient ovary (primary site) from which OCI-C5x was derived, stained for S100A1 to identify ovarian tumor cells from normal ovarian epithelial cells. Regions marked with "N" indicate necrotic areas that are false positive for S100A1 staining. The boxes indicate the ten regions chosen for *ERBB2*:CEP17 scoring by FISH. Scale bar 5mm. (B) Higher magnification of Region #3 (S100A1). Scale bar 1mm (C) Representative image of FISH staining for *ERBB2* (red) and CEP17 (green), representative images from one of three independent experiments. Scale bar 20μm. (D) IHC of ERBB2 staining of Region #3. Scale bar 1mm. (E) Summary table of the *ERBB2*:CEP17 patterns in all the tested regions. Percentage of scored cells with *ERBB2*:CEP17 equal or greater than two are shown and *ERBB2*:CEP17 signal ratio was calculated for this group of cells. Cells with only one CEP17 signal and two ERBB2 signals were excluded from this analysis to reduce false positives.

Supplementary Figure 6.

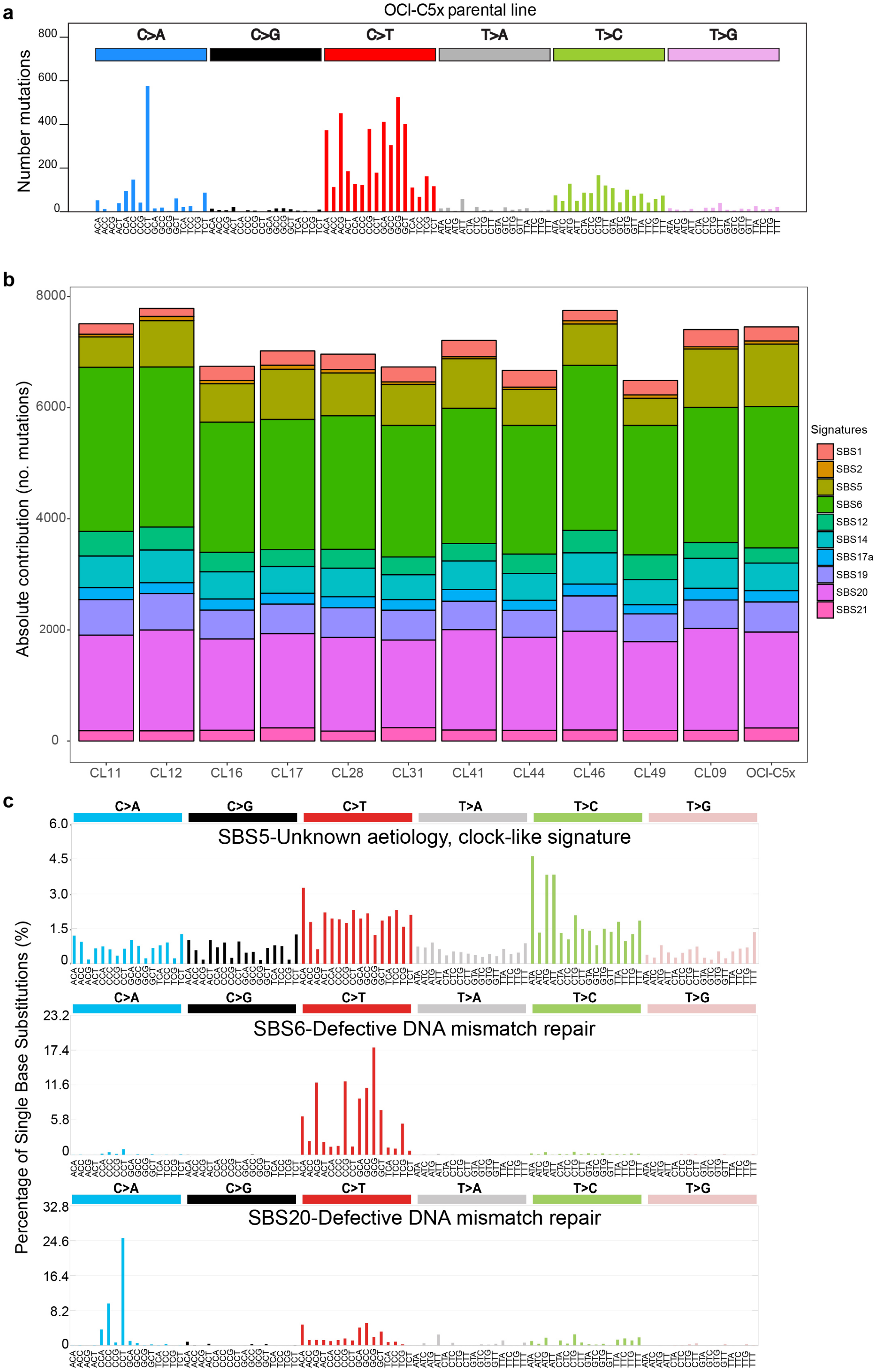

Supplementary Figure 6: Mutational signatures and allele frequency analyses.

(A) Spectrum of 96 trinucleotide mutational contexts for 7,484 filter-passing mutations in the parental line. The six substitution types are shown above, and the bases immediately 5' and 3' to the mutated base are shown below. (B) Comparison of Single Base Substitution (SBS) mutational signatures present in each clone. No significant differences between the samples. (C) Reference Cosmic SBS signatures v 3.1 for the three most active signatures in the samples. Proposed aetiology listed, if available, see (https://cancer.sanger.ac.uk/cosmic/signatures/SBS) for further detail.

Supplementary Figure 7.

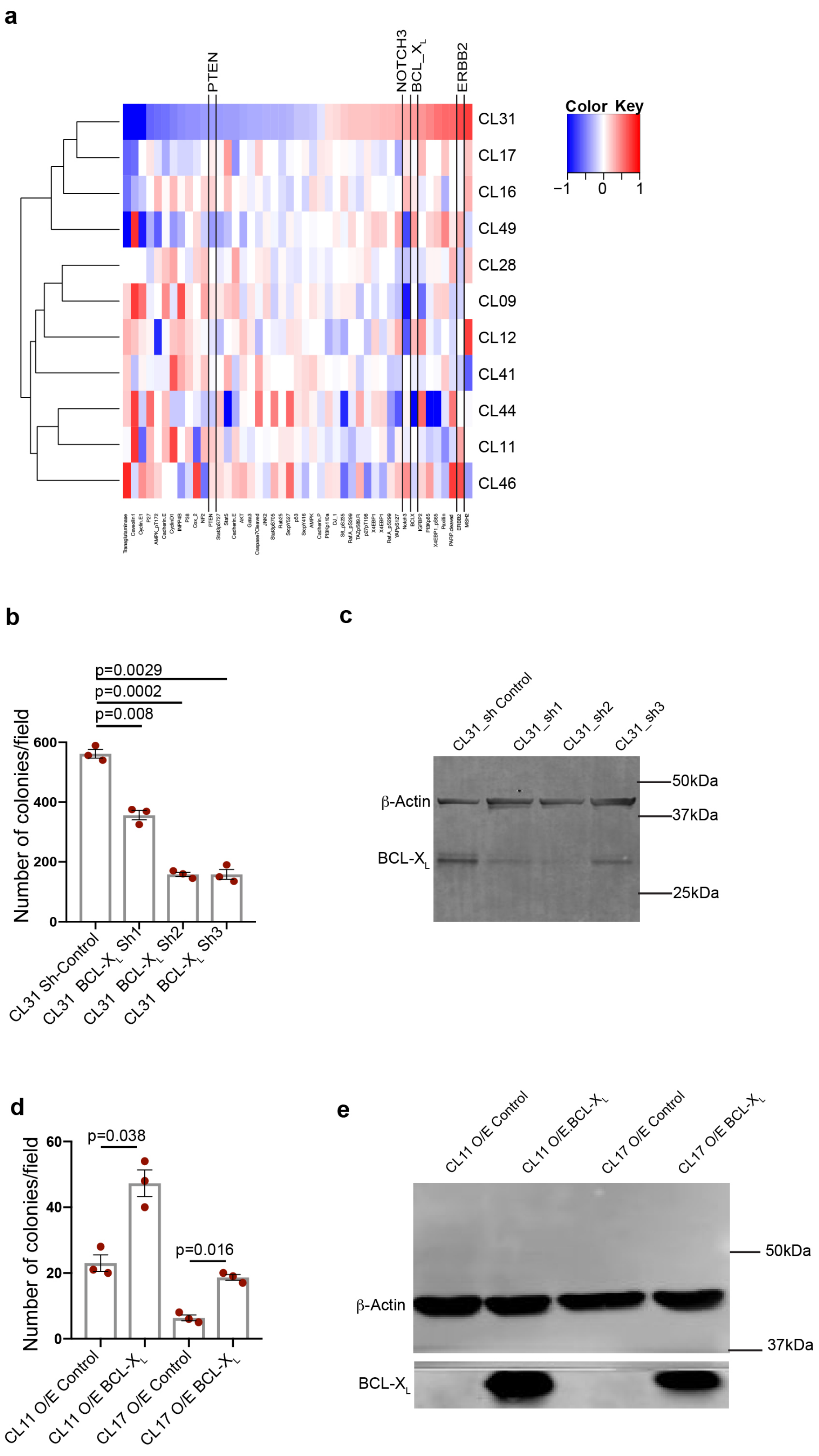

Supplementary Figure 7: Differentially expressed proteins in CL31

(A) RPPA analysis of clonal populations. Differentially expressed proteins in CL31 relative to the other clonal populations are shown (unpaired two-tail Student's t test $p < 0.05$). Heatmap represents the average of triplicate samples (log2-transformed and median-centered). (B) Quantification of colony formation in soft agar by CL31 expressing a control shRNA or one of three distinct BCL-X_L shRNAs. The number of colonies were counted per field and averaged for each condition. Three independent experiments were summarized by mean $\pm$ SEM. *p* values were computed using the one-way ANOVA test and corrected for multiple comparisons using Dunnett's method. (C) Western blot showing BCL-X_L knockdown in CL31 using three distinct shRNAs, representative images from one of three independent experiments (D) Quantification of number of colonies formed by either CL11 or CL17 overexpressing ERBB2 and western blot showing ERBB2 levels in control and ERBB2 overexpressing clones. The number of colonies were counted per field and averaged for each condition. Data shown as mean $\pm$ SEM of three independent experiments, *p* value from Welch's t test of the means. (E) Western blot showing BCL-X_L expression in the engineered lines, representative images from one of three independent experiments.

Supplementary Figure 8.

a

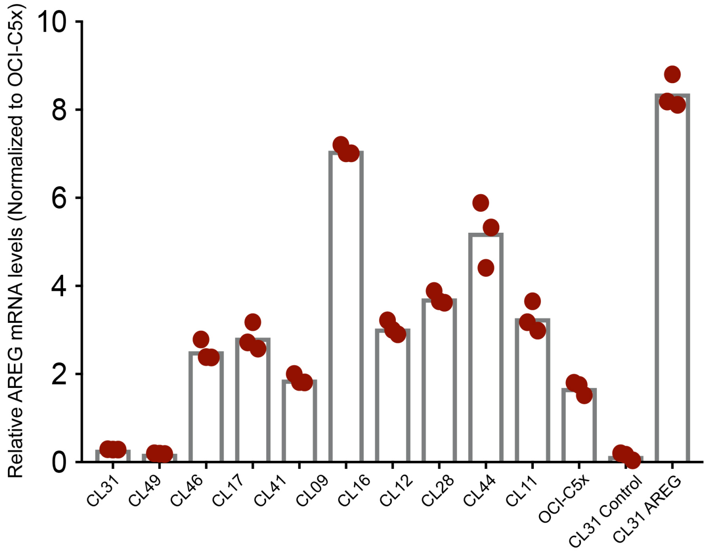

b

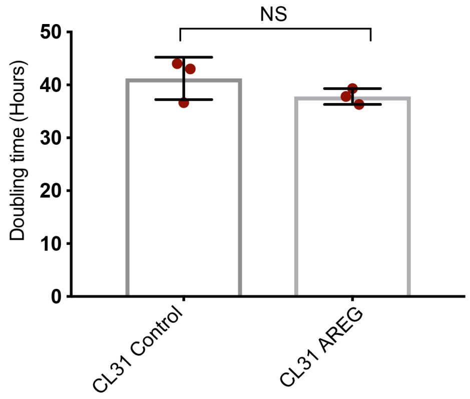

Supplementary Figure 8: AREG overexpression in CL31.

(A) qRT-PCR measurements of mRNA levels of AREG in the OCI-C5x parental line, the clonal populations and CL31 transduced with empty vector (Control) or AREG. Measurements from three technical replicates were normalized to *RPLPO* mRNA levels and expressed as fold change compared to the OCI-C5x parental line. (B) Doubling time of CL31-Control and CL31-AREG lines grown in triplicate and for over 5 days. Doubling time was computed as follows: 24*[(LOG (cell number day 1)-LOG (cell number day 5))/LOG(2)]. Data shown as mean ± SEM of three independent experiments, *P* value from Welch's t test of the means. NS: not significant.

Supplementary Figure 9.

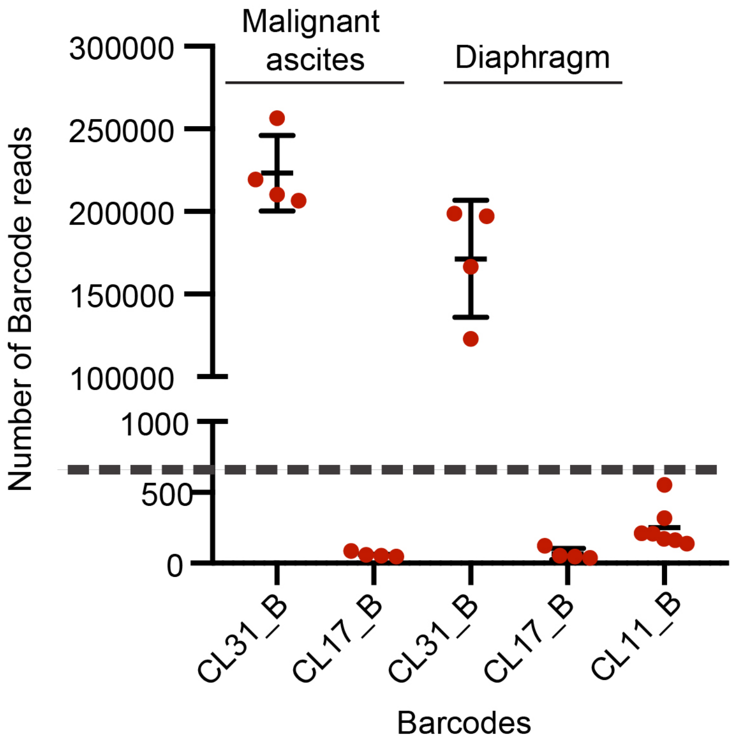

Supplementary Figure 9: Malignant ascites and solid peritoneal metastases generated by the CL31:CL17 mixture consist exclusively of CL31.

CL31 and CL17 barcodes counts (CL31_B, CL17_B, respectively) of indicated samples collected from mice at 10 weeks following IP injection of CL31:CL17 mixture. The barcode count for CL11, which was not present in the mixture, was used as a negative control to set the noise threshold of the barcode counts (dashed line). Data presented as mean ± SD from one experiment (n=4 of each group).

Supplementary Figure 10.

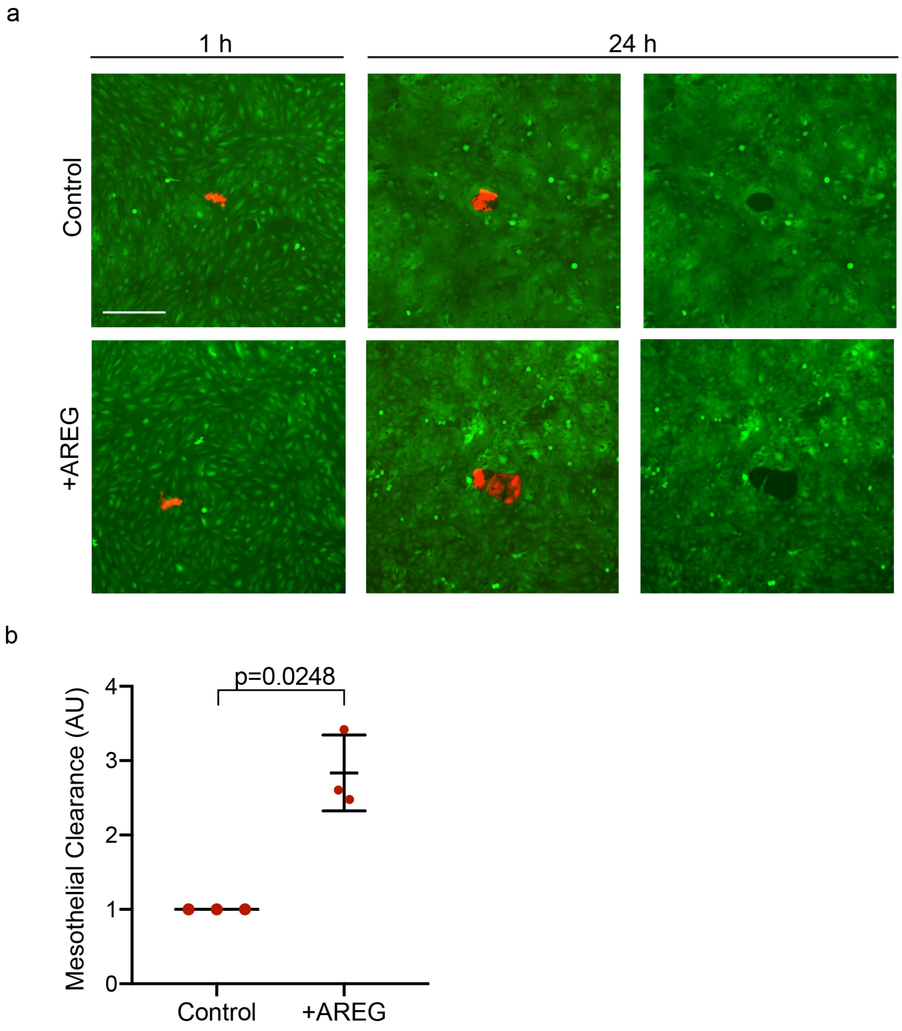

Supplementary Figure 10: AREG enhances mesothelial clearance ability of CL49

(A) Representative fluorescence images of ability of vehicle- or AREG-treated CL49 cell clusters (red) to clear a mesothelial monolayer (green) at the indicated time points. Scale bar, 200μm. (B) Quantification of the mesothelial clearance area (black area within the green monolayer) cleared in 24 hours by CL49 spheroids treated with vehicle or AREG from three independent experiments. Mesothelial area cleared at the endpoint was normalized to the initial (1h) area of CL49 clusters. Relative clearance area of 20-30 clusters of CL49 of each condition per experiment were analyzed and averaged. Data shown as mean ± SEM of three independent experiments, shown in arbitrary units (AU), *p* value from Welch's t test of the means.

Supplementary Figure 11.

a

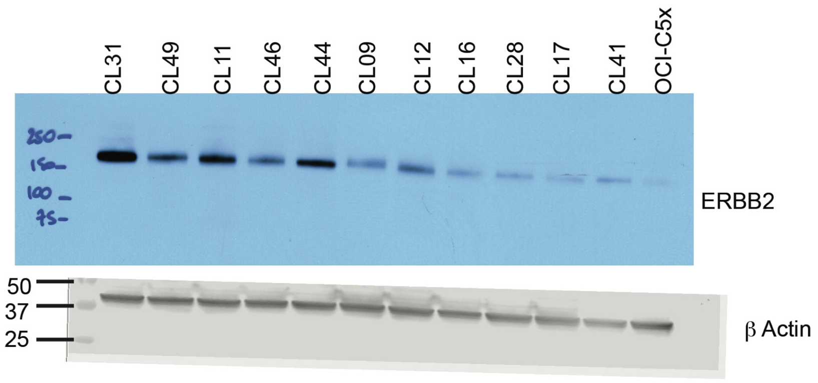

b

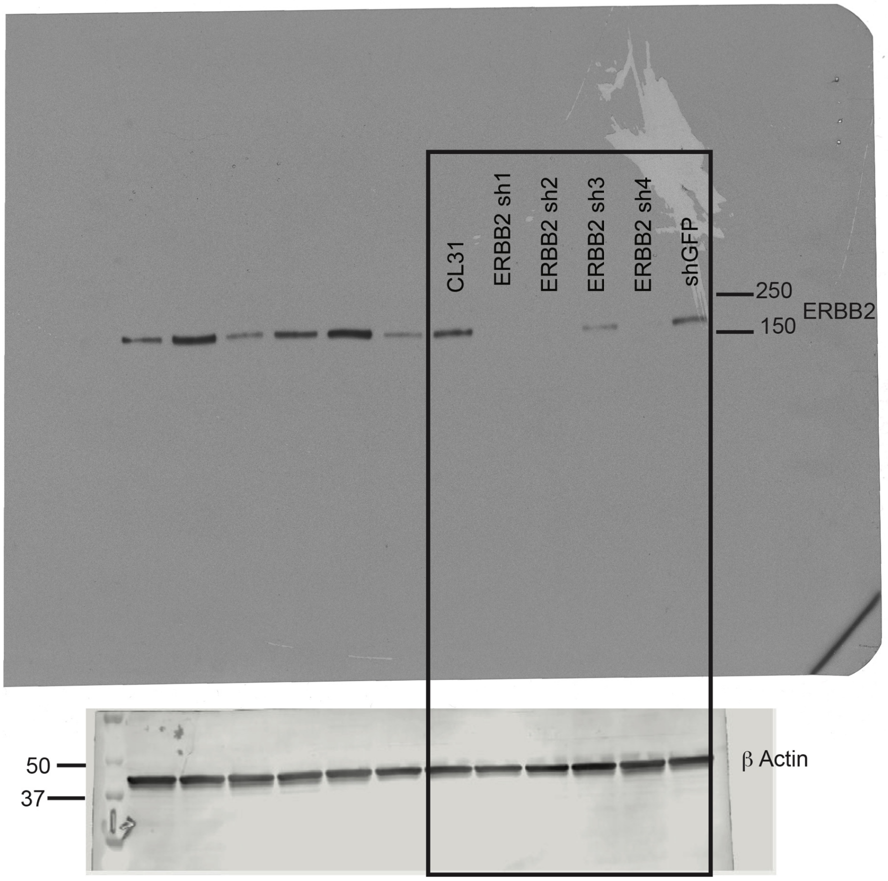

Supplementary Figure 11: Full scan for blots in Figure 3.
Western blot scans or figure 3e (A) and 3g (B).

Supplementary Figure 12.

a

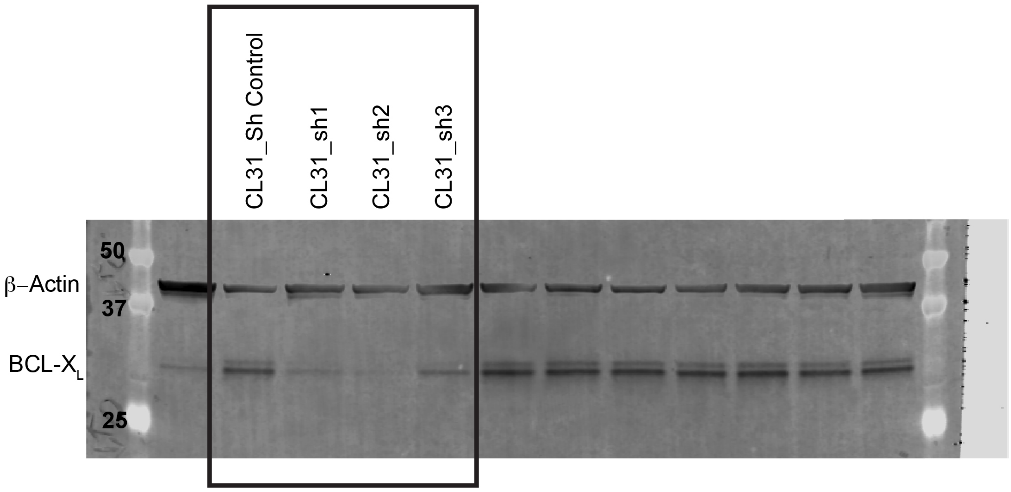

b

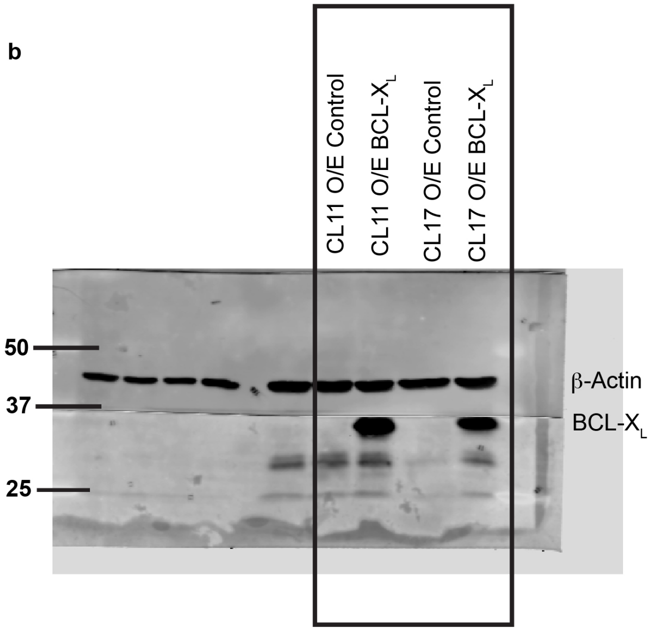

Supplementary Figure 12: Full scan for blots in supplementary Fig. 7.
Western blot scans for Supplementary Fig. 7c (A) and Supplementary Fig. 7e (B).

Supplementary Table 1: Barcodes ID and their target clones

Clone_ID	Barcode_ID	Barcode Sequence
CL09	WS-01-SingleWS	TGACTGTGAGTGTCTGTCACAGTGTGTGAG
CL44	WS-08-SingleWS	TGTGTCTGACTGACTGACAGTGACACACTG
CL49	WS-21-SingleWS	TCTGTGTCACACACTCTGTCTGAGAGTGTC
CL17	WS-17-SingleWS	TGACTCAGAGAGTCTCTGTGTCTGTCAGAC
CL41	WS-23-SingleWS	ACTGTCTGAGACAGAGAGTGTGACAGTCAG
CL16	WS-16-SingleWS	TCTCTGAGACACAGTCAGAGTCACAGTGTG
CL46	WS-19-SingleWS	AGACAGACTCTCAGTCTGTCAGACAGTGAG
CL31	WS-14-SingleWS	AGTGTCACTGTGTGACTGAGAGTCTGACAG
CL11	WS-03-SingleWS	TCTGACACTCAGACTCAGTGACTGTGACTG
CL28	WS-13-SingleWS	TCAGTCTCAGTCTCACTGTGTGTCACTCTC
CL12	WS-04-SingleWS	TGACAGAGTGTGTCAGAGTGTGAGTGAGTG

Supplementary Table 2: Primers sequences

Primer Name	Primer Sequence
WSL_NGS_Barcode_Seq	GCGACCACCGAGATCTACACACTGACTGCAGTC TGAGTCTGACAG
WSL_NGS_Index_Seq	GATCGGAAGAGCACACGTCTGAACTCCAGTCAC
Amphiregulin Forward primer	TGATCCTCACAGCTGTTGCT
Amphiregulin Reverse primer	TCCATTCTCTTGTCGAAGTTTCT
RPLPO Forward primer	ACGGGTACAAACGAGTCCTG
RPLPO Reverse primer	CGACTCTTCCTTGGCTTCAA
