# Supplementary material for: Transient commensal clonal interactions can drive tumor metastasis
Source: Nat Commun. 2020 Nov 16;11:5799. doi: 10.1038/s41467-020-19584-1 (PMC7669858; doi:10.1038/s41467-020-19584-1)
Supplement: Supplementary file 2 — Description of Additional Supplementary Files [file 41467_2020_19584_MOESM2_ESM.docx]

**File Name: Supplementary Data 1**

Description: Raw barcode counts and distribution analyses (related to Figure 2)

**File Name: Supplementary Data 2**

Description: List of mutations detected by whole-exome sequencing (related to Figure 3f and Supplementary Figure 6)
