# Supplementary material for: Transient commensal clonal interactions can drive tumor metastasis
Source: Nat Commun. 2020 Nov 16;11:5799. doi: 10.1038/s41467-020-19584-1 (PMC7669858; doi:10.1038/s41467-020-19584-1)
Supplement: Supplementary file 5 — Reporting Summary [file 41467_2020_19584_MOESM5_ESM.pdf]

nature research

Corresponding author(s): Joan S. Brugge

Last updated by author(s): Oct 20, 2020

Reporting Summary

Nature Research wishes to improve the reproducibility of the work that we publish. This form provides structure for consistency and transparency in reporting. For further information on Nature Research policies, see Authors & Referees and the Editorial Policy Checklist .

Statistics

For all statistical analyses, confirm that the following items are present in the figure legend, table legend, main text, or Methods section.

n/a	Confirmed	
☐	☒	The exact sample size (*n*) for each experimental group/condition, given as a discrete number and unit of measurement
☐	☒	A statement on whether measurements were taken from distinct samples or whether the same sample was measured repeatedly
☐	☒	The statistical test(s) used AND whether they are one- or two-sided<br>*Only common tests should be described solely by name; describe more complex techniques in the Methods section.*
☒	☐	A description of all covariates tested
☐	☒	A description of any assumptions or corrections, such as tests of normality and adjustment for multiple comparisons
☐	☒	A full description of the statistical parameters including central tendency (e.g. means) or other basic estimates (e.g. regression coefficient) AND variation (e.g. standard deviation) or associated estimates of uncertainty (e.g. confidence intervals)
☐	☒	For null hypothesis testing, the test statistic (e.g. *F*, *t*, *r*) with confidence intervals, effect sizes, degrees of freedom and *P* value noted<br>*Give P values as exact values whenever suitable.*
☒	☐	For Bayesian analysis, information on the choice of priors and Markov chain Monte Carlo settings
☒	☐	For hierarchical and complex designs, identification of the appropriate level for tests and full reporting of outcomes
☒	☐	Estimates of effect sizes (e.g. Cohen's *d*, Pearson's *r*), indicating how they were calculated

Our web collection on statistics for biologists contains articles on many of the points above.

Software and code

Policy information about availability of computer code

Data collection

Software was not used for data collection, only analysis.

Data analysis

All data analyses were performed using publicly available software:
For RNAseq analysis: RNAseq analysis was performed in R.3.5.1. Reads were mapped to the hg19 version of the human genome using bowtie2-2.1.0. Quantification of counts per gene was performed using HTSeq-0.5.4 and differential expression was performed with EdgeR-3.24.3. Hierarchical clustering was performed using Spearman correlation and average linkage. Heat maps were generated with the heatmap.2 function of gplot-3.0.3.
Functional annotation of mutation calls from the whole exsome sequencing data were conducted using Oncotator (http://portals.broadinstitute.org/oncotrator/)
For Tree building: mpboot v.1.1.0 open source program was used.
Whole-exome sequencing was aligned using BWA-MEM
Mutations were called using Mutect v 1.1.4
Mutational patterns R package v 2.0.0 (https://github.com/UMCUGenetics/MutationalPatterns)
For Copy number variation analysis: DNA sequences were aligned to HG19 using Bowtie2 and genomic copy number was calculated at 220kb resolution using variable binning and CBS segmentation. The resulting copy number profiles were used for hierarchical clustering and annotated using the cancer gene census (Futreal 2004).
Statistical analysis were performed using Prism 7.0 software.
Image analysis were performed using ImageJ 1.48V
Flow cytometry graphs were prepared using flowJo v7.6.5

For manuscripts utilizing custom algorithms or software that are central to the research but not yet described in published literature, software must be made available to editors/reviewers. We strongly encourage code deposition in a community repository (e.g. GitHub). See the Nature Research guidelines for submitting code & software for further information.

Data

Policy information about availability of data

All manuscripts must include a data availability statement. This statement should provide the following information, where applicable:

- Accession codes, unique identifiers, or web links for publicly available datasets
- A list of figures that have associated raw data
- A description of any restrictions on data availability

The data that supports the findings of this study are included in this published article (and its supplementary information files), and have been deposited in Gene Expression Omnibus (GEO), GSE123426 (RNAseq data). The CNA has been deposited into the Sequence Read Archive (SRA), accession ID: PRJNA658185. Both data are publicly available. The WES data has been deposited into the Sequence Read Archive (SRA), accession ID: PRJNA668770. All the data are publicly available.

Field-specific reporting

Please select the one below that is the best fit for your research. If you are not sure, read the appropriate sections before making your selection.

- [x] Life sciences
- [ ] Behavioural & social sciences
- [ ] Ecological, evolutionary & environmental sciences

For a reference copy of the document with all sections, see nature.com/documents/nr-reporting-summary-flat.pdf

Life sciences study design

All studies must disclose on these points even when the disclosure is negative.

Sample size	For in vitro experiments, we included at least 3 biological replicates, each including 3 technical replicates. For most in vivo experiments, 5 mice were included in each biological replicate. We did not perform power analysis prior performing to these experiments, rather we chose these sample sizes based on a pilot experiment using 5 mice in each group to compare metastatic outgrowth for two single clonal populations (CL31 and CL49) and multi clonal mixture. This experiment demonstrated that five mice were sufficient to detect a significant difference (p= p=0.0134) between the mixture and the individual clones. One experiment was carried out with 3 mice (figure 5e), we chose this number based on a pilot experiment which detected a significance difference with 3 mice (p value=0.0029). Therefore we included 3-5 mice in each experiment (as described) and carried out at least 2 biological replicates.
Data exclusions	One Barcode experiment was excluded due to contamination of other barcodes used in the lab..
Replication	The reproducibility of the experiments was confirmed using at least three biological replicates in all in vitro experiments. For the in vivo experiments, at least two biological replicates were performed.<br>The clonal representation experiments (Figure 2f) were carried out successfully and with consistent results three times, but we had to exclude the barcode representation analysis of one of the experiments due to contamination of other barcodes used in the lab.
Randomization	All mice used in the experiments were randomly allocated into experiment groups prior to any treatments/procedures.
Blinding	The investigator who recorded the tumor burden scores was blinded. For the other experiments, where the readouts were quantitative, blinding was not considered.

Reporting for specific materials, systems and methods

We require information from authors about some types of materials, experimental systems and methods used in many studies. Here, indicate whether each material, system or method listed is relevant to your study. If you are not sure if a list item applies to your research, read the appropriate section before selecting a response.

Materials & experimental systems

n/a	Involved in the study	
[ ]	[x]	Antibodies
[ ]	[x]	Eukaryotic cell lines
[x]	[ ]	Palaeontology
[ ]	[x]	Animals and other organisms
[ ]	[x]	Human research participants
[x]	[ ]	Clinical data

Methods

n/a	Involved in the study	
[x]	[ ]	ChIP-seq
[ ]	[x]	Flow cytometry
[x]	[ ]	MRI-based neuroimaging

Antibodies

Antibodies used	All antibodies used in the figures are listed in the Methods with supplier name and catalog number.<br>Anti ERBBE for IHC staining: anti-ERBB2 antibody (Dako, A0485), dilution 1:200<br>S100A1 antibody (Dako, Z031129-2), dilution 1:500

For western blot: Anti-ERBB2 Thermo Fisher Scientific MS325-P1, dilution 1:500.
Anti BCL-XL, Cell Signaling 2764S, dilution 1:500.
beta-Actin, Cell Signaling 3700S, dilution 1:5000.
For flow cytometry: Anti-ERBB2 primary antibody pre-conjugated with secondary antibody (Brilliant Violet) (Biolegend, 324420) , dilution 1:100.

Validation	S100A1 antibody has been validated by immunohistochemistry as indicated on the manufacturer's website. ERBB2 antibody for IHC has been validated by immunohistochemistry staining as indicated on the manufacturer's website, and has been used in our laboratory. In addition, positive control tissue (Her2 positive breast cancer tissue) was used for additional validation. BCL-XL, ERBB2 and beta Actin for western blot were validated for western blotting as indicated on the manufacturer's website, and has been used previously by our laboratory.<br>For flow cytometry, ERBB2 antibody pre-conjugated with secondary antibody has been validated by immunohistochemistry as indicated on the manufacturer's website and has been in use in our laboratory.<br>Amphiregulin blocking antibody was developed and extensively validated by Dr. Yarden's lab as published in Carvalho S. et al, Oncogen, 2016. The activity of Amphiregulin blocking antibody was also confirmed in our laboratory by western blot to validate its efficiency in suppressing downstream signaling (pERK1/2) in a dose dependent manner.

Eukaryotic cell lines

Policy information about cell lines

Cell line source(s)	OCI-C5x cell line, Prof. Tan Ince, Weil Cornell School of Medicine
Authentication	STR was performed for authentication purposes.
Mycoplasma contamination	Cells were tested negative for mycoplasma contamination
Commonly misidentified lines (See ICLAC register)	No misidentified lines were used

Animals and other organisms

Policy information about studies involving animals; ARRIVE guidelines recommended for reporting animal research

Laboratory animals	Nod SCID Gamma mice, 8-10 weeks old, Femaless. Animals were housed in HMS animal facility, in temperatures 20-22c, humidity 30-70% and a 12-hour light/12-hour dark cycle.
Wild animals	This study did not involve wild animals
Field-collected samples	This study did not involve samples collected from the field.
Ethics oversight	IACUC at Harvard Medical School, protocol no. IS00990

Note that full information on the approval of the study protocol must also be provided in the manuscript.

Human research participants

Policy information about studies involving human research participants

Population characteristics	NA
Recruitment	NA
Ethics oversight	NA

Note that full information on the approval of the study protocol must also be provided in the manuscript.

Flow Cytometry

Plots

Confirm that:

- [x] The axis labels state the marker and fluorochrome used (e.g. CD4-FITC).
- [x] The axis scales are clearly visible. Include numbers along axes only for bottom left plot of group (a 'group' is an analysis of identical markers).
- [x] All plots are contour plots with outliers or pseudocolor plots.
- [x] A numerical value for number of cells or percentage (with statistics) is provided.

Methodology

Sample preparation	Cells were cultured in OCMI medium and harvested with Trypsin-EDTA during log phase growth. Cells were washed twice with1x PBS and blocked with 5% goat serum in PBS. For ERBB2 stain, Anti-ERBB2 primary antibody pre-conjugated with secondary antibody (Brilliant Violet) (Biolegend, 324420) was used (1:100).
Instrument	FACSCalibur (BD Biosciences, Inc).
Software	Flow cytometry data was analyzed using FlowJo v7.6.5 (FlowJo LLC)
Cell population abundance	The flow cytometry experiment included in our manuscript was used to sort out cells expressing the highest and lowest levels of ERBB2. Unstained control was used as a reference sample to gate for the positive population for ERBB2. Next, we sorted OCI-C5x for subpopulations with high ERBB2 (highest 5%) and low ERBB2 (lowest 5%) expression.
Gating strategy	OCI-C5x unstained control was used to define negative population in the OCI-C5x-Erbb2 stained population. Subpopulations with high ERBB2 (highest 5%) and low ERBB2 (lowest 5%) expression were sorted.

☐ Tick this box to confirm that a figure exemplifying the gating strategy is provided in the Supplementary Information.
